# Zinc finger proteins (ZFPs) in health and disease

**DOI:** 10.1186/s43556-026-00413-8

**Published:** 2026-03-03

**Authors:** Zhenxin Zhao, Kairan Huang, Zi Liao, Bei Chen, Jing Chen, Zhigang Mei

**Affiliations:** 1 School of Medicine and Health Sciences, China Three Gorges University, No.8, Daxue Road, Xiling District, Yichang, Hubei 443002 China; 2 Academy of Chinese Medical Sciences, Hunan University of Chinese Medicine, No.300, Xueshi Road, Yuelu District, Changsha, Hunan 410208 China; 3 Key Laboratory of Hunan Province for Integrated Traditional Chinese and Western Medicine On Prevention and Treatment of Cardio-Cerebral Diseases, Hunan University of Chinese Medicine, Changsha, Hunan 410208 China

**Keywords:** Zinc finger protein, Cancer, CNS and genetic disorders, Autoimmune diseases, Viral infections

## Abstract

Zinc finger proteins (ZFPs), a vast superfamily of sequence-specific DNA and RNA-binding proteins, serve as master regulators of gene expression and cellular homeostasis. While traditionally studied for their roles in development, ZFPs have emerged as critical effectors and therapeutic targets across a wide spectrum of human pathologies, including cancer, neurological disorders, and autoimmune diseases. This review systematically dissects the molecular mechanisms by which dysregulated ZFP activity drives disease pathogenesis, using ischemic stroke as a central exemplar to illustrate their multifaceted roles. We detail how specific ZFPs orchestrate key stroke risk factors such as hypertension, hyperglycemia, and atherosclerosis, subsequently govern post-ischemic injury cascades, including neuroinflammation, programmed cell death, and blood–brain barrier disruption. Addressing the long-standing challenge of ZFPs as “undruggable” targets, we critically evaluate cutting-edge therapeutic strategies poised to modulate their function with precision. These include small-molecule modulators, targeted protein degraders (PROTACs), zinc finger nuclease (ZFN)-based gene editing, and advanced nanocarrier delivery systems, complemented by high-throughput computational screening. By integrating deep mechanistic insights with novel translational approaches, this review establishes a pioneering pan-disease framework for targeting ZFP networks. We provide a structured roadmap for future research and highlight the immense potential of ZFPs as a new class of master regulatory targets for developing novel and feasible therapies in ischemic stroke and beyond.

## Introduction

Zinc finger proteins (ZFPs) constitute one of the largest and most functionally diverse superfamilies of transcriptional regulatory factors in eukaryotes and exhibit widespread distribution across diverse biological kingdoms, including animals, plants, and microorganisms [1–3]. As pivotal regulatory molecules, ZFPs recognize nucleic acids in a sequence-dependent manner and interact with proteins through structurally defined interfaces, thereby orchestrating fundamental cellular processes including gene expression, chromatin remodeling, and signal transduction [4]. Since the initial identification of the zinc finger domain, this superfamily has expanded significantly with the continuous discovery of diverse members, among which the C2H2 type represents the most abundant and extensively studied subclass. Accumulating evidence highlights the dual biological significance of ZFPs in maintaining homeostasis and driving pathogenesis. Under physiological conditions, ZFPs dictate cell-autonomous processes, including differentiation, development [5], DNA damage repair [6], and RNA metabolism [7], while simultaneously orchestrating systemic immune surveillance and metabolic equilibrium. Conversely, the perturbation of ZFP expression or functional integrity often precipitates pathological transitions. Specific ZFPs function as proto-oncogenes or tumor suppressors in cancer [8, 9], whereas their dysfunction underlies neuronal attrition and proteostatic collapse in central nervous system (CNS) disorders [10, 11]. Furthermore, ZFPs critically modulate host immune responses during autoimmune disorders and viral infections [12, 13]. Consequently, ZFPs sit at the nexus of cellular physiology and disease, where the balance of their regulatory networks determines the ultimate biological outcome.

Mechanistic studies have delineated fundamental aspects of ZFPs function, including their domain architecture, DNA-binding specificity, and contributions to chromatin remodeling and transcriptional complexes. However, critical questions regarding the context-dependent biology of ZFPs persist. It remains unclear how individual ZFPs achieve target gene specificity and execute disparate functional outcomes across diverse cell types, developmental stages, and pathological conditions. Moreover, how the complex interplay within ZFP networks, including cooperative binding, competitive inhibition, and hierarchical regulation, integrates into cohesive transcriptional programs that govern cellular fate is not yet fully understood. A significant knowledge gap also exists concerning the mechanisms by which ZFP activities are incorporated into broader signaling pathways to orchestrate pivotal cell fate decisions, such as the balance between survival and apoptosis in stroke or between proliferation and quiescence in cancer. Addressing these unresolved issues is imperative, as the pleiotropic and context-specific nature of ZFPs continues to present a formidable challenge to the design of precise and effective therapeutic interventions.

In this review, we provide a comprehensive overview of ZFP structure and classification. We then systematically evaluate their roles in both physiological and pathological contexts, with a focused discussion on their pathogenic contributions across major human disease domains. These include prominent cancers of the lung, liver, breast, and colorectum; a spectrum of CNS disorders spanning stroke, Alzheimer’s disease (AD), Parkinson’s disease (PD), amyotrophic lateral sclerosis, epilepsy, and depression; genetic disorders; autoimmune diseases; and viral infections. Furthermore, we examine recent advances in ZFP-targeted therapeutic strategies, encompassing small molecule modulators, protein degradation technologies, and gene-editing platforms. Through the integration of structural, functional, and disease-oriented perspectives, the therapeutic potential of ZFP networks across diverse pathological contexts is highlighted. Unlike earlier reviews that frequently focus on isolated ZFP subfamilies or single disease models [14, 15], we present an integrated framework linking fundamental molecular mechanisms to pan-disease pathophysiology. Consequently, this review aims to decipher context-specific ZFP regulatory logic, identify actionable nodes for translational research, and delineate a path toward next-generation therapies in oncology, neurology, genetics, and immunology by leveraging targeted ZFP modulation.

## Zinc finger protein family: classification and structure

**Fig. 1:**
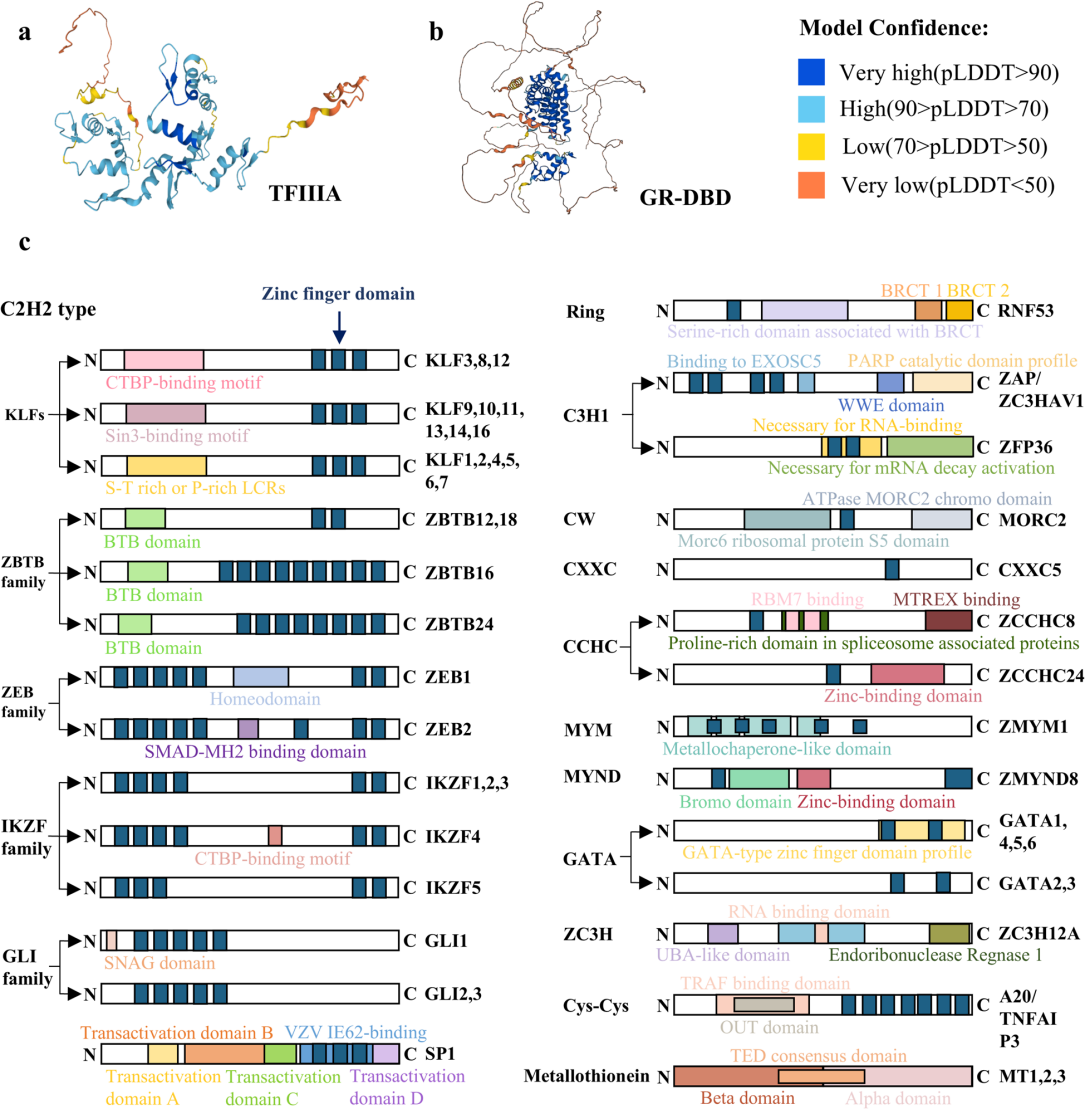
The protein structure of ZFP family **a** Domain structure of ZFPs. **b** Three dimensional structures of TFIIIA, Glucocorticoid receptor DNA binding domain (GR-DBD) from AlphaFold (TFIIIA: Cys₂His₂ type;GR-DBD: Cys₄ type). **c** Domain of ZFP family

ZFPs constitute a distinct class of proteins characterized by the presence of zinc finger domains, which are structurally stabilized through the coordination of zinc ions (Zn^2^⁺) [16]. These proteins are encoded by approximately 5% of the human genome [17], and exhibit a broad distribution across animal [3], plant [2], and microbial species [1]. The core structure of ZFPs is defined by a looped region comprising approximately 30 amino acids. Within this region, Zn^2^⁺ are stably incorporated into the structural framework through two classical coordination modes: the Cys₂His₂ type, where tetrahedral coordination bonds are formed by the sulfur and nitrogen atoms of two cysteine (Cys) residues and one histidine (His) residue (as illustrated in Fig. 1a), and the Cys₄ type, which relies on the concerted coordination of zinc ions by the sulfur atoms of four cysteine residues (as shown in Fig. 1b). The coordination bonds between Zn^2^⁺ and amino acid residues enable the protein backbone to adopt a stable ββα fold, conferring a "finger-like" three-dimensional conformation that serves as the critical structural basis for the specific recognition of DNA/RNA or proteins by ZFPs. Among these, TFIIIA exemplifies the functional mechanism of ZFPs as transcriptional regulators by binding to the 5S ribosomal RNA gene promoter through its zinc finger array and recruiting RNA polymerase III, thereby providing a classical paradigm for the role of ZFPs in transcriptional regulation [18].

To facilitate the systematic investigation of ZFPs, researchers have developed a hierarchical classification framework rooted in their intrinsic structural features. This taxonomy primarily relies on two fundamental criteria: first, the quantitative variations in coordination bonds formed by Cys and His residues, which prompted the HUGO Gene Nomenclature Committee (HGNC) to delineate 30 distinct subtypes of non-canonical zinc fingers in 2015 [19]; and second, the stereochemical configuration of these residues around the Zn^2^⁺ ion. Based on the three-dimensional organization of their zinc-binding domains, characterized ZFPs have been systematically categorized into eight structural families: Cys₂His₂ (C₂H₂)-like, Gag knuckle, Treble clef, zinc ribbon, Zn₂/Cys₆, TAZ2 domain-like, short zinc-binding loops, and Metallothionein-related motifs [20]. These proteins are widely disseminated across diverse cellular types and tissues. They primarily act as transcription factors, being localized within either the nucleus or cytoplasm, with certain members even possessing the capability to traverse between the cytoplasm and nucleus [21].

Following this discovery, scientists have unveiled an extensive array of zinc-binding structures, which not only facilitate protein–protein interactions [22], but also bind to RNA [23], ubiquitin, SUMO, methylated histones, and poly(ADP-ribose) [3]. To date, the ZFP family comprises over 50 unique structural domains [24]. This structural diversity underpins their broad functional versatility, allowing ZFPs to participate in a multitude of fundamental cellular processes. This functional versatility also underscores the significant pathological consequences of ZFP dysregulation or mutation, a feature summarized in Table 1 across diverse diseases including cancer, CNS disorders, genetic disorders, autoimmune diseases, viral infections.

**Table 1:** Classification of ZFPs and their functional roles in health and disease

Protein Types	Family members	Targets or pathways	Underlying Mechanism	Diseases	Ref
C2H2	KLF2	TM, eNOS, PAI-1, BDNF/TrkB, TJs	Maintaining endothelial homeostasis, antithrombotic, anti-microglial apoptosis, protecting the BBB	Atherosclerosis, IS	[25–28]
	KLF4	IL-1β, TGF-β1, EEPD1, NF-κB, Nrf2/Trx1, FTO, STAT3	Anti-inflammation, anti-neuronal apoptosis, promoting angiogenesis, protecting the BBB, promoting synaptic plasticity	Lung cancer, IS, atherosclerosis, epilepsy	[29–33]
	KLF5	LXRα, CXCL12	Stimulating renin secretion, impairing of vascular repair	Hypertension, hyperglycemia	[34, 35]
	KLF6	SV1 and SV2, Nrf2/HO-1, HSP47	Anti-cell proliferation, promote apoptosis and ferroptosis	Lung cancer, IS, epilepsy	[36–38]
	KLF11	PPARγ, occludin, ZO-1	Inhibition of EC apoptosis, protecting the BBB	IS	[39, 40]
	KLF13	SM22α	Promoting VSMCs phenotypic dedifferentiation	Atherosclerosis	[41]
	ZEB1	53BP1, LCN2, GPX4, NuRD, PHGDH, β-Catenin, AK2/STAT3/STAT4, COX-2, EMT, VCAM1 and ICAM1, TGF-β1, TAp73	Promoting NHEJ and inhibiting HR, promoting or inhibiting ferroptosis and inflammation, promoting tumorigenesis and metastasis, β-cell function and survival	Lung cancer, breast cancer, CRC, HCC, MS, ASL, atherosclerosis, hyperglycemia, IS	[42–50]
	ZEB2	SIP1, CD274 and CCL2, E-cadherin, EMT, JAK-STAT, Notch and TGF-β1	Immunosuppression, promoting tumorigenesis and metastasis, regulating SMC phenotypic transition	Lung cancer, MWS, SLE, atherosclerosis	[51–54]
	ZBTB16/PLZF	WDHD1, CD4 TRM, GRK2/HIF-1α, AT2R	Promoting neural differentiation, inhibiting DNA replication and inducing cell cycle arrest, anti-angiogenic, inhibiting neuronal apoptosis	lung cancer, RA, RSV infection, IS	[55–58]
	ZBTB18	ID1-4, Ngn2	Neuronal generation and maturation	CRC	[59, 60]
	ZBTB24	CDCA7	Promoting DNA methylation homeostasis	ICF syndrome	[61]
	IKZF1	PI3K/AKT	Promoting T-cell and medullary thymic epithelial cell homeostasis,	SLE	[62, 63]
	IKZF2	FOXP3	Promoting T-cell homeostasis	RA	[64]
	IKZF3	CD62L	Regulating B-cell differentiation and lymphocyte homing	MS	[65]
	ZFP24	VEGF-A, MMP2	Promoting angiogenesis, promoting tumorigenesis and metastasis	NSCLC, CRC	[66, 67]
	ZFP42/MZF1	P53	Inhibiting cell proliferation	Breast cancer	[68]
	ZFP64	Sema3A, ENO2/HK2, GCH1 and FTH1	Promoting developmental climbing fiber synapse elimination, tumorigenesis, triggering immune evasion	Breast cancer, TNBC, HCC	[69–72]
	ZFP90	TLR4-PI3K-AKT-NF-κB	Chronic inflammation and gut microbiota dysbiosis	CRC	[73]
	ZFP131	RAD51, PAIP1, SMC4	Promoting HR and cell proliferation	NSCLC, HCC	[74–76]
	ZFP148	RXRα	inhibiting HBV replication	HBV	[77]
	ZFP207	U1 snRNA, ENO1 and GAPDH	promoting spliceosome assembly, inhibiting aerobic glycolysis	HCC	[78, 79]
	ZFP274	SNORD116	Organising 3D genomic structures	PWS	[80, 81]
	ZFP277	P21waf1	regulating cellular proliferation and senescence	CRC	[82]
	ZFP280A	RPS14	inhibiting proliferation and tumorigenicity	CRC	[83]
	ZFP281	XRCC4, GDNF and NRP2, TGF-β1	Promoting NHEJ, inhibiting neuronal differentiation, regulating colon fibroblast activation and myofibroblast differentiation	CD	[84–86]
	ZFP335	REST/NRSF, Hadha, Lmnb1	Promoting neural and T cell development	Autosomal recessive microcephaly	[87–90]
	ZFP384/ZER6	Ku70/Ku80, ZEB1, MMP2, POLR3G	Organising 3D genomic structures, promoting cNHEJ, promoting tumorigenesis and metastasis	Breast cancer, CRC, NSCLC	[91–94]
	ZFP398	MT3	Increasing reactive oxygen species	AD	[95]
	ZFP427	Htr2a	promoting stress resilience	Depression	[96]
	ZFP498	P53	Inhibiting apoptosis and ferroptosis	HCC	[97]
	ZFP652	CCND3, PD-L1	Downregulating cyclin D3, immune evasion	Lung cancer, TNBC	[98, 99]
	ZFP667	LDH	Anti-oxidative stress	IS	[100]
	ZFP689	LINE-1	Promoting intratumor heterogeneity	TNBC	[101]
	ZFP695	NEK2	Promoting proliferation	CRC	[102]
	ZFP764	PGC-1α, NRF2	Promoting oxidative stress and apoptosis	AD, PD	[103, 104]
	ZFP795/SALL2	AXIN2	Promoting apoptotic	CRC	[105]
	ZFP801/MAZ	CCND1, KRAS, TYMP	Regulating the cell cycle, promoting tumor proliferation and immune evasion	Lung cancer, HCC	[106, 107]
	ZFP802/JAZF1	PI3K-Akt-AMPK	Inhibiting hepatic glucose production	Hyperglycemia	[108]
	ZFP831	Wnt/β-catenin, JAK/STAT	promoting tumorigenesis and metastasis, apoptosis	Lung cancer, breast cancer	[109, 110]
	REST/NRSF	Aβ and tau, Kv7.2/7.3 potassium channels	Inhibiting Aβ and tau, cognitive Impairment	AD, epilepsy	[111, 112]
	YY1	TREM2, Fuzzy	Promoting Aβ, inducing synaptic deficits	AD, ALS	[113, 114]
	CTCF	PARP-1	Organising 3D genomic structures, promoting HR	Neurodevelopmental disorders	[115–117]
	GLIS3	Ins2, MafA	Regulating insulin expression and β cell function	Hyperglycemia	[118]
	SP1	TIGAR, NCX1, ACSL4	Anti-oxidative stress, promoting ferroptosis	IS	[119, 120]
	MTF-1	NCX1	Anti-oxidative stress	IS	[121]
	Gli 1	Shh pathway	Anti-oxidative stress	IS, epilepsy	[122, 123]
RING	BRCA1/RNF53	53BP1	Promoting HR	Cancer	[124]
C3H1	ZFP36	3'UTR, CCND1, HDAC3, RGS2, CEMIP	RNA degradation and regulation of T-cell homeostasis, reducing cyclin D1 expression, promoting neointimal hyperplasia	CRC, hypertension, atherosclerosis	[125–129]
	ZAP/ZC3HAV1	KHNYN, STING	RNA degradation, promoting immune inflammation	Viral infection	[130–132]
CW	MORC2	RBM39, CDK5RAP2	Orchestrating chromatin remodelling and DNA repair, oxidative stress and mitochondrial dysfunction	Breast cancer, CRC, CMT2Z	[133–135]
CXXC	CXXC5	NuRD, TSC1/mTOR	Regulating chromatin remodelling, promoting tumorigenesis	Breast cancer	[136]
CCHC	ZCCHC8	IRF3	Promoting RNA virus replication	RNA virus infection	[137]
	ZCCHC24	ZEB1	Inhibiting tumor growth	TNBC	[138]
MYM	ZMYM1	RAS/ERK/c-FOS, E-cadherin	Promoting tumorigenesis and metastasis	HCC	[139]
MYND	ZMYND8	ZEB1, HK2	Promoting tumorigenesis and metastasis	Breast cancer, HCC, autosomal dominant neurodevelopmental disorder	[140–142]
GATA	GATA1	IL-1β and TNF-α	Promoting inflammation	Depression	[143]
	GATA2	IFITM1	Promoting proliferation, migration and macrophage-like transdifferentiation of VAMCs	Atherosclerosis	[144]
	GATA6	ACE2, PDGF	Inhibiting neointimal formation	SARS-CoV-2 viral infection, atherosclerosis	[145, 146]
CCCH(ZC3H)	MCPIP1	JNK/NF-κB, TFRC/AKT/mTOR, MMP, TJs	Anti-inflammation, promoting angiogenesis, protecting the BBB	IS	[147–149]
Cys-Cys	A20	NF-κB, RIPK3	Anti-inflammation, inhibiting neuronal apoptosis and necroptosis	IS	[150–152]
Metallothionein	MTIII	8-OHdG, Zn2 +, superoxide anions	Anti-oxidative stress	IS	[153]

*IS* ischemic stroke, *NSCLC* non-small cell lung cancer, *TNBC* triple-negative breast cancer, *CRC* colorectal cancer, *HCC* hepatocellular carcinoma, *AD* Alzheimer's disease, *PD* Parkinson's disease, *ALS* amyotrophic lateral sclerosis, *MS* multiple sclerosis, *MWS* Mowat‒Wilson syndrome, *RSV* Respiratory syncytial virus, *RA* rheumatoid arthritis, *ICF syndrome* immunodeficiency, centromeric instability, and facial anomalies syndrome, *SLE* systemic lupus erythematosus, *PWS* Prader-Willi syndrome, *CD* Crohn's disease, *CMT2Z* Charcot-Marie-Tooth disease type 2Z, *BBB* blood–brain barrier, *EC* endothelial cell, *VSMCs* vascular smooth muscle cells, *NHEJ* non-homologous end joining, *HR* homologous recombination, *Aβ* β-amyloid

## Physiological roles of ZFPs

ZFPs constitute a core regulatory network across multiple biological tiers by virtue of their specific nucleic acid-binding properties. At the molecular level, ZFPs integrate transcriptional regulation, DNA damage repair, and RNA metabolism to ensure precise coordination of genetic information flow. These coordinated functions collectively determine cellular fate, thereby driving tissue differentiation and organismal development. At the systemic level, ZFPs orchestrate immune cell differentiation and function while establishing the molecular foundations for immune tolerance, thereby underpinning robust organismal defense and maintaining systemic homeostasis.

### Gene expression regulation

Gene expression represents a fundamental biological process directed by transcription factors (TFs), which operate by recognizing and binding specific DNA sequences through diverse regulatory mechanisms [154]. As a major class of transcription factors, ZFPs primarily operate within the nucleus, where they recognize and bind to specific DNA sequences through their canonical zinc finger domains. This interaction directly modulates gene expression, either through transcriptional activation, as exemplified by ZFP143 at mitochondrial gene promoters [155], or through repression, such as the inhibition of nerve growth factor (NGF) promoter activity by ZFP662 [156]. Beyond canonical DNA recognition, certain ZFPs recognize non-B-form DNA structures, such as G-quadruplexes bound by MYC-associated zinc finger protein (MAZ), or prevent transcription-replication conflicts, as demonstrated for ZC3H4, thereby enabling transcriptional regulation through non-canonical pathways [106, 157].

Beyond direct transcriptional control, ZFPs contribute to programming stable, long-term gene expression patterns via epigenetic modulation. In this capacity, they serve as molecular adaptors that couple DNA recognition with alterations to the chromatin landscape. For example, ZFP545 was reported to suppress transcription by recruiting the KRAB-associated protein 1 (KAP1) complex to catalyze repressive H3K9me3 histone marks [158], while ZFP331 functioned as a scaffold for co-repressors, including tripartite motif-containing 28 (TRIM28) and histone deacetylases to establish a silenced chromatin environment [159]. Furthermore, proteins such as zinc finger E-box binding homeobox 1 (ZEB1) and CXXC-type zinc finger protein 5 (CXXC5) modulate chromatin remodeling and accessibility by interacting with the nucleosome remodeling and deacetylase (NuRD) complex [136, 160]. ZFPs also influence nucleic acid modifications, as exemplified by ZBTB24, which was found to regulate DNA methylation patterns by controlling downstream target genes [61].

Furthermore, ZFPs function as pivotal architects of the three-dimensional (3D) genome. The canonical example, CTCF, defines the boundaries of topologically associated domains (TADs) and establishes chromatin loops, thereby shaping the genomic spatial framework to ensure precise enhancer-promoter communication [115, 117]. Beyond the foundational role of CTCF, other ZFPs contribute to 3D genome organization through distinct mechanisms. For instance, ZFP143 directly mediates the formation of specific chromatin loops to facilitate cooperative gene cluster transcription [161]; ZFP384 localizes to TAD boundaries to maintain structural stability while supporting high-level transcription [91], and ZFP274 orchestrates spatial segregation and sustained repression by tethering gene clusters to nucleolus-associated domains [80].

In summary, ZFPs are master integrators of gene regulation. By converging sequence-specific DNA recognition, epigenetic modification, and the orchestration of 3D genome architecture, they function as indispensable nodes within comprehensive gene regulatory networks. Importantly, ZFPs maintain genomic integrity not only by regulating gene expression but also through their active participation in the fundamental DNA damage response. Precisely this functional crossover between gene control and genome protection ensures precise genetic transmission and cellular homeostasis, offering a key lens through which to understand the comprehensive role of ZFPs in health and disease.

### DNA repair and damage response

**Fig. 2:**
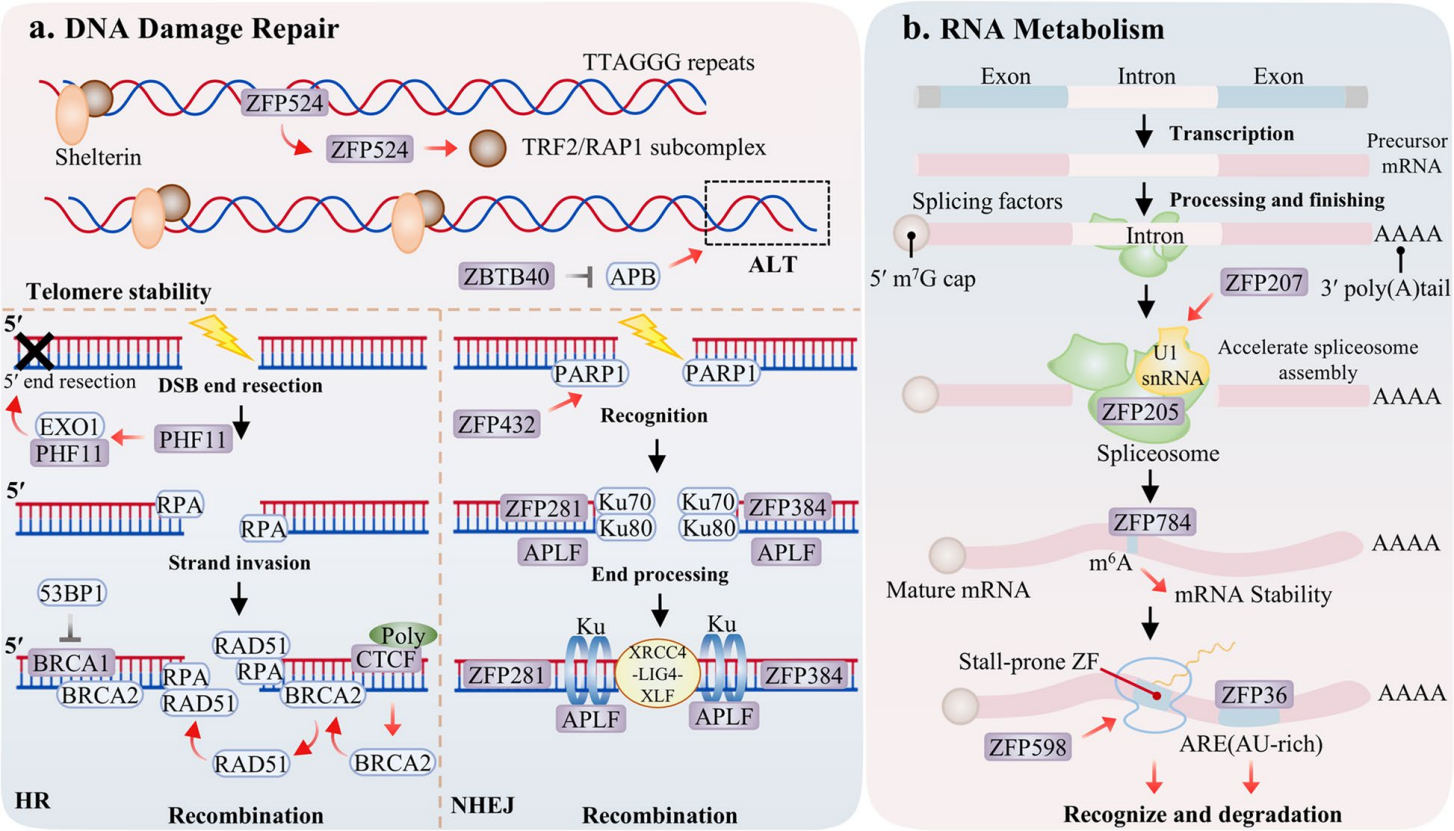
Roles of ZFPs in nucleic acid metabolism and regulation. ZFPs ensure cellular function by coordinating DNA stability with RNA metabolic control. In the context of DNA, ZFPs contribute to genomic integrity through a dual mechanism: they maintain telomere stability to prevent damage initiation (e.g., ZFP524, ZBTB48) and, in response to DNA double-strand breaks, function as scaffold proteins (e.g., APLF, CTCF) or direct facilitators of repair complex assembly (e.g., ZFP281, ZFP384, ZFP432, BRCA1) to enable accurate restoration of the genome. In parallel, within RNA metabolism, ZFPs perform critical post-transcriptional functions by directly modulating pre-mRNA splicing efficiency (e.g., ZFP207); recognizing RNA modifications to alter transcript stability (e.g., ZFP784); and actively controlling mRNA translation and turnover through mechanisms such as binding AU-rich elements, as performed by the ZFP36 family, or by monitoring translational fidelity (e.g., ZNF598)

DNA double-strand breaks (DSBs) represent one of the most cytotoxic forms of DNA damage. Their erroneous repair can precipitate mutations and chromosomal rearrangements, which are hallmarks of carcinogenesis [162]. Eukaryotic cells have evolved two principal pathways to resolve DSBs: the rapid but error-prone non-homologous end joining (NHEJ) and the high-fidelity, template-dependent homologous recombination (HR) [163]. ZFPs have emerged as pivotal regulators in this process, orchestrating the assembly of repair complexes, directing pathway choice, and modulating local chromatin structure to ensure faithful genome repair (Fig. 2a).

Significantly, the role of ZFPs in preserving genomic integrity extends beyond direct DSB repair to encompass proactive genome maintenance. A principal example is the safeguarding of telomere stability, which prevents the natural ends of chromosomes from being erroneously identified as DSBs, thereby precluding inappropriate activation of the DNA damage response. For instance, ZFP524 directly binds to telomeric repeat sequences to suppress local DNA damage signaling [164]. Similarly, in cells that utilize alternative lengthening of telomeres (ALT), ZBTB40 binds to telomeric DNA to prevent aberrant elongation and preserve telomere function [165].

When DSBs arise internally within the genome, ZFPs are pivotal in executing and coordinating the downstream repair responses. Within the NHEJ pathway, numerous ZFPs serve as either core components or critical regulatory factors, facilitating the direct ligation of broken DNA ends. For example, ZFP281 and ZFP384 directly promote the assembly of the NHEJ repair complex through specific interactions with the core components X-ray repair cross complementing 4 (XRCC4) and Ku70/Ku80, respectively [84, 166]. Aprataxin and PNKP like factor (APLF), in contrast, functions as a scaffold protein, recruiting and stabilizing the XRCC4 DNA ligase IV (Lig4)/XRCC4-like factor (XLF) ligation complex via its von willebrand factor A domain binding to Ku80 [167, 168]. Furthermore, ZFP432, acting as a poly (ADP ribose) reader, not only stimulates poly (ADP-ribose) polymerase 1 (PARP1) activity but also suppresses HR, thereby actively modulating the pathway choice between NHEJ and HR [6].

ZFPs similarly play vital roles in the HR pathway, participating in processes ranging from initial DNA end resection to the recruitment of core RAD51 homolog 1 (RAD51). PHF11, for example, facilitates end resection by alleviating replication protein A (RPA) mediated inhibition of exonuclease 1 (EXO1), thereby generating the 3’ single stranded DNA overhang essential for RAD51 loading [169]. Breast cancer type 1 susceptibility protein (BRCA1), which contains a RING zinc finger domain, is a pivotal factor promoting RAD51 recruitment to damage sites [124]. Meanwhile, CTCF functions as a scaffold protein that recruits breast cancer type 2 susceptibility protein (BRCA2) in a PARylation-dependent manner to facilitate BRCA2-RAD51 complex formation [116]. Concurrently, CTCF leverages its capacity for 3D genome organization to prevent repair signal diffusion and maintain the spatial specificity of repair events [170].

ZFPs actively regulate the critical choice between DNA repair pathways. For instance, RNF138 promotes HR by disassembling the Ku complex [171], whereas ZEB1 exerts opposing regulation by inhibiting HR and reinforcing NHEJ, with studies revealing its potential as a biomarker for PARP inhibitor efficacy in BRCA1-deficient cancers [42].

At the post-translational modification level, ZFPs serve as key executors of DNA damage signaling. RNF168 catalyzes ubiquitination of histones H2A/H2AX, creating a platform for 53BP1 and BRCA1 recruitment to amplify DNA damage response signals [172]. Concurrently, ZFP451, functioning as a SUMO E3 ligase, catalyzes SUMO2 modification of RNF168, which stabilizes its protein levels and enhances its accumulation at damage sites [172].

In summary, ZFPs function as integral guardians of genomic integrity, operating across a continuum from upstream prevention to downstream repair. They operate as central molecular hubs and epigenetic coordinators, precisely regulating repair pathway selection and efficiency through protein interactions, modification recognition, and chromatin remodeling. Simultaneously, ZFPs engage in preventive maintenance, such as telomere stabilization, to preemptively suppress illegitimate DNA damage signaling. However, their notable functional plasticity, exemplified by ZEB1 and ZFP432’s dual regulation of both NHEJ and HR balance, presents significant challenges for targeted therapeutic strategies. Therefore, future research should prioritize elucidating their dynamic regulatory mechanisms within specific cellular and pathological contexts. It is noteworthy that the maintenance of genetic information stability by ZFPs is intimately connected to the subsequent processes of its expression and utilization. The functions of numerous ZFPs further extend into the post-transcriptional realm, where they assume pivotal roles in RNA metabolism and fate determination, thereby achieving global regulation of the genetic information flow.

### RNA metabolism and regulation

RNA metabolism encompasses a highly coordinated multistep process, including transcription, processing, translation, and degradation. Within this framework, ZFPs function not only as classical transcription factors but also as master regulators of post-transcriptional RNA homeostasis. Systems biology studies have revealed that knockdown of numerous ZFPs leads to widespread transcriptome dysregulation, underscoring their universal importance in maintaining RNA metabolic balance (Fig. 2b) [7].

At the post-transcriptional level, ZFPs prominently regulate alternative splicing of pre-mRNA, accomplishing this through multiple distinct strategies. For example, ZFP207 accelerates spliceosome assembly by binding to U1 snRNA, thereby enhancing splicing efficiency [78]. Concurrently, ZRANB2/ZFP265 functions as an intrinsic spliceosome component, directly recognizing specific RNA sequences to modulate splicing outcomes [173].

Furthermore, ZFPs extensively participate in RNA modification, stability control, and translational regulation. They contribute to transcriptomic regulation, exemplified by ZFP784, which interprets N6 methyladenosine (m⁶A) modifications to influence mRNA stability [174]. Regarding stability control, CCCH type ZFPs, including members of the ZFP36 family, promote rapid mRNA degradation by binding to AU rich elements in 3’ untranslated regions [125]. Notably, ZFP36L2 directly binds to flavivirus RNA via its CCCH motif and degrades viral RNA, relying on the 5′ 3’ XRN1 pathway [175].

The terminal step of RNA metabolism, targeted degradation, is precisely executed by several ZFPs. ZFP598 is responsible for degrading mRNAs associated with stalled ribosomes [176]. Similarly, ZCCHC family members mark RNAs for degradation or participate directly in decay complexes [177]. The antiviral protein zinc-finger antiviral protein (ZAP) specifically recognizes and directs the degradation of foreign RNAs [130].

Notably, ZFPs do not function in isolation but form complex regulatory networks. For instance, ZFP36L1 inhibits vascular smooth muscle cells (VSMCs) proliferation by degrading the mRNA of another ZFP, KLF16, thereby establishing hierarchical regulation among ZFPs [178]. These networks effectively integrate RNA metabolism with broader cellular physiological processes to maintain cellular homeostasis.

In summary, RNA fate is regulated by ZFPs through mechanisms that include sequence-specific RNA binding, modification recognition, and protein–protein interactions. Their coordinated actions establish ZFPs as central regulators of RNA metabolism. This role, when integrated with their direct DNA-binding at the transcriptional level, constitutes the foundational mechanism for establishing complex gene expression programs.

### Cell differentiation and development

Building upon their deep regulatory control over gene expression and RNA metabolism, ZFPs further function as central orchestrators of cellular differentiation and developmental programs. They direct cell fate decisions, tissue patterning, and organ formation by precisely controlling the spatiotemporal expression of specific gene networks. This role is exemplified during the complex and highly ordered process of nervous system development, where ZFP activity is essential throughout, from neural progenitor fate specification to terminal neuronal maturation and circuit assembly.

During the early stages of neural development, neural progenitor cells (NPCs) undergo rapid proliferation, followed by differentiation into neurons or glial cells at specific developmental timepoints. This process is critically orchestrated by numerous ZFPs. For instance, ZFP521 was shown to activate neurogenic gene expression by synergizing with p300, thereby guiding embryonic stem cells toward NPCs fate [5]. ZBTB16 and ZFP335 were identified as essential for maintaining proper NPCs states, with ZBTB16 driving proliferation [55] and ZFP335 ensuring cell cycle progression through binding key promoter regions of factors such as REST/NRSF [87]. Concurrently, ZFP536 and ZFP281 were found to finely control neuronal numbers by delaying or inhibiting differentiation [85, 179]. Regarding neuronal subtype specification, ZFP238 functions as a transcriptional repressor that establishes identity and hierarchical positioning of subcortical projection neurons [59], while factors including specificity protein 9 (SP9) are indispensable for normal development of striatal projection neurons [180]. During neuronal morphological maturation and functional refinement, ZFPs maintain critical functions. ZFP189 was shown to promote the mature morphology of dendritic spines in pyramidal neurons, a process closely linked to higher-order neural functions [181].

Beyond the nervous system, ZFPs serve as global developmental regulators across multiple tissues and organs. In cell lineage commitment, zinc finger E-box binding homeobox 1 (ZEB2) drives pluripotent stem cell differentiation into myogenic progenitor cells [182], while ZFP423 regulates mesenchymal stem cell differentiation into adipocytes [183]. During organ morphogenesis, the ZEB2-encoded smad interaction protein 1 (SIP1) protein coordinates retinal neuron differentiation and lens fiber cell alignment [51]. For systemic homeostasis maintenance, Kruppel-like factor 2 (KLF2) and Kruppel-like factor 4 (KLF4) protect vascular endothelial health by responding to blood flow shear stress [25]. Furthermore, during terminal erythroid maturation, Kruppel-like factor 1 (KLF1) and ZFP410 respectively regulate nuclear expulsion and fetal hemoglobin expression to ensure functional red blood cell generation [184, 185].

In summary, ZFPs constitute a multi-layered regulatory network. This network precisely coordinates cell fate determination, tissue patterning, and organ formation, thereby orchestrating developmental and differentiation processes across biological systems.

### Immune regulation

The immune system relies on stringent regulation of cellular development, differentiation, activation, and tolerance. These processes are governed by multi-layered gene expression programs. Within this framework, ZFPs function as critical regulatory molecules, shaping immune cell fate and function through transcriptional control, epigenetic modification, metabolic reprogramming, and signal transduction.

In adaptive immunity, ZFPs serve as core transcriptional regulators. They precisely modulate lymphocyte lineage differentiation and functional homeostasis. Members of the Ikaros family, including Ikaros family zinc finger (IKZF) 1 and IKZF3, establish essential transcriptional networks for lymphocyte differentiation via heterodimer formation and recruitment of chromatin-modifying complexes [186]. In T cells, IKZF1 prevents excessive activation by inhibiting the phosphatidylinositol 3-kinase (PI3K)/protein kinase B (AKT) signaling pathway [187], while ZFP335 maintaines peripheral T cell homeostasis through regulation of Lmnb1 transcription [89]. IKZF3 similarly governs B cell development and lymphocyte homing processes [188]. It also establishes activation thresholds by balancing B cell receptor signaling components [189]. Additionally, ZBTB20 maintains immune homeostasis in myeloid immune cells, such as dendritic cells [190].

Within innate immunity, ZFPs participate directly in antiviral defense and inflammatory regulation. ZAP specifically recognizes viral RNA and recruits cellular cofactors to form antiviral complexes, thereby restricting viral replication during early infection [131]. Similarly, ZAP enhances inflammation by binding with stimulator of interferon genes (STING) and promoting its oligomerization and translocation [132]. Meanwhile, ZEB2 guides macrophage polarization through synergistic binding with specificity protein 1 (SP1) at gene promoter [52].

In immune tolerance establishment, ZFPs provide the molecular foundation for self-tolerance mechanisms. IKZF1 ensures proper self-antigen presentation and clonal deletion in the thymus by regulating thymic epithelial cell development [62]. In the periphery, IKZF2 maintains regulatory T cell (Treg) suppressive function by regulating forkhead box P3 (FOXP3) expression via epigenetic mechanisms [191]. Furthermore, ZFP36 family members stabilize Treg function through post-transcriptional degradation of specific mRNAs [126]. Concurrently, ZFP335 supports effector Treg differentiation in early life by regulating the fatty acid oxidation (FAO) enzyme Hadha [88].

In summary, ZFPs function as indispensable regulators within the immune system through their integrative capabilities across pathways and cell types. Their coordinated actions establish and maintain immune homeostasis, while their dysfunction contributes to immune-related pathologies. Elucidating ZFP-mediated mechanisms provides crucial molecular insights and identifies potential therapeutic targets for immune disorders.

Collectively, the functions of ZFPs span a biological hierarchy from DNA damage repair and genomic stability to the regulation of genetic information flow via gene expression and RNA metabolism, and further extend to directing cell fate through development and differentiation, as well as maintaining organismal defense via immune regulation. This multifaceted involvement across molecular, cellular, and organismal levels firmly establishes ZFPs as pivotal hub proteins capable of integrating complex biological networks. Importantly, the dysfunction of these regulatory hubs is central to their transition from homeostatic guardians to drivers of pathology.

## ZFPs in diseases

Dysfunction of ZFPs constitutes a common molecular underpinning driving the initiation and progression of human diseases. As core transcriptional regulators, ZFPs form disease-specific pathological networks across various disorders: in cancer, they drive malignant tumor progression by modulating the cell cycle, metabolic reprogramming and the immune microenvironment; in CNS disorders, they mediate neuroinflammation, programmed cell death and synaptic plasticity impairment; in genetic diseases, as direct effectors of genetic defects, they cause developmental anomalies and functional deficits; in autoimmune diseases, they dynamically participate in the breakdown of immune tolerance, inflammatory cell differentiation and tissue damage; and in viral infections, they establish defensive systems by directly interfering with viral replication and regulating host immune responses. These functions highlight ZFPs as key regulatory nodes in a broad spectrum of pathological processes.

### Cancer

In disease states, particularly in malignant tumors, the tightly regulated functions of ZFPs are frequently disrupted, thereby driving tumorigenesis, progression, metastasis and therapeutic resistance. By modulating key biological processes including cell proliferation, metabolic reprogramming, epithelial-mesenchymal transition (EMT) and immune evasion, ZFPs synergistically promote tumor progression.

#### Lung cancer

Lung cancer remains the leading cause of cancer-related mortality worldwide [192]. Non-small cell lung cancer (NSCLC) constitutes its most common pathological subtype. The ZFP family has been demonstrated to play a pivotal role in the initiation and progression of this disease, functioning through a sophisticated regulatory network. This network modulates core biological processes, including cell proliferation, metastasis, metabolism, and the tumor microenvironment.

In the context of cell proliferation, ZFPs influence tumor growth primarily through cell-cycle regulation. For example, ZFP652 acts as a tumor suppressor by inhibiting cyclin D3, thereby inducing G1 phase arrest [98], while ZBTB16 interferes with WD repeat and HMG-box DNA binding protein 1 (WDHD1) transcription, which leads to S phase arrest [8]. Similarly, within the Wnt/β‑catenin pathway, ZEB1 promotes proliferation by upregulating wnt family member 7B (WNT7B) gene, whereas ZFP831 inhibites it by suppressing YTH N6-methyladenosine RNA binding protein 1 (YTHDF1) gene [9, 109]. Members of the KLF family display functional diversity. KLF4 suppresses growth, while its splice variant KLF6-SV1 facilitates disease progression [36]. During EMT and metastasis, ZEB1 and ZEB2 function as central regulators. Their expression is modulated by miR-200c, collectively establishing a critical axis that influences metastatic potential and drug resistance [53]. Furthermore, microenvironmental factors, such as lactic acid secreted by cancer-associated fibroblasts, upregulate ZFP384. This enhances RNA polymerase III subunit G (POLR3G) transcription and subsequently induces EMT [94].

Therapeutic resistance in NSCLC has been closely associated with DNA damage repair and metabolic reprogramming mediated by ZFPs. ZFP131 drives tumor progression and therapeutic resistance by binding to the RAD51 promoter and enhancing homologous recombination repair [74]. Metabolically, ZEB1 suppresses ferroptosis and promotes proliferation via upregulation of lipocalin 2 (LCN2). Additionally, its stability, augmented by FBXO protein 45 (FBXO45), further facilitates the Warburg effect and inhibits ferroptosis [43, 193]. In tumor microenvironment remodeling, ZFPs contribute to angiogenesis and immune regulation. For instance, MAZ induces the expression of immunosuppressive molecules, including Gal-9, through KRAS activation. This establishes an immunosuppressive microenvironment conducive to immune evasion [107]. Separately, SRY-box transcription factor 4 (SOX4) triggers VEGF-A secretion by downregulating ZFP24, which promotes angiogenesis [66].

In summary, ZFPs constitute a multifunctional and highly interconnected regulatory network in lung cancer. Core nodes, such as ZEB1, have been implicated concurrently in EMT, ferroptosis resistance, and proliferative signaling [9, 43]. The functions of these proteins exhibit heterogeneity across lung cancer subtypes. For example, MAZ drives proliferation and immune evasion in lung adenocarcinoma [107, 194], whereas ZFP367 operates through the Hippo/YAP pathway in small cell lung cancer [195]. These findings suggest that reactivating such proteins may represent a promising therapeutic strategy. Elucidating this intricate network not only advances the mechanistic understanding of lung cancer but also offers new perspectives and molecular targets for the development of precision therapies tailored to specific pathological contexts.

#### Breast cancer

Breast cancer is the most prevalent malignant tumor among women worldwide, having surpassed lung cancer as the most frequently diagnosed cancer [192]. Its development and progression involve numerous regulated biological processes. Within these processes, ZFPs constitute a complex regulatory network, modulating key mechanisms such as the cell cycle, apoptosis, EMT, metabolic pathways, and the tumor microenvironment.

In the context of malignant proliferation, ZFPs exhibit bidirectional control over cell fate. For instance, ZEB1 promotes mitosis through the epigenetic suppression of phospholipase D3 (PLD3), which subsequently relieves inhibition of cyclin-dependent kinase 1 (CDK1) [196]. Conversely, ZFP831 facilitates apoptosis by inhibiting the signal transducer and activator of transcription 3 (STAT3)/B-cell lymphoma-2 (Bcl2) axis [110], and myeloid zinc finger 1 (MZF1) exerts tumor-suppressive effects by enhancing p53 acetylation [68]. During invasion and metastasis, ZEB1 functions as a central regulator of EMT. It enhances cellular motility and stemness through suppression of epithelial markers and the miR-200 family [197]. The ubiquitin specific protease (USP) 7–MYND-type zinc finger-containing chromatin reader (ZMYND8) axis modulates its expression and activity [140]. Additionally, ZFP384 forms a positive feedback loop with ZEB1, continuously reinforcing the invasive phenotype [92].

Metabolic reprogramming and DNA repair processes are critical for tumor adaptation and therapeutic resistance. ZFP64 drives the Warburg effect by directly activating glycolytic genes [70]. ZEB1 also plays a central role by influencing metabolism, redox balance, and autophagy through upregulation of glycolytic enzymes and modulation of glutathione peroxidase 4 (GPX4) [44, 198]. At the level of DNA repair, MORC2 mediates chemotherapy resistance by enhancing SUMOylation modifications [133]. Within the tumor microenvironment and immune regulation, ZEB1 induces M2-type macrophage polarization via lactate, thereby establishing an immunosuppressive microenvironment [198]. Separately, CXXC5 promotes immune evasion by inhibiting the tuberous sclerosis complex subunit 1 (TSC1)/mammalian target of rapamycin (mTOR) pathway, resulting in upregulation of programmed cell death-ligand protein 1 (PD-L1) [136].

In the more aggressive triple-negative breast cancer (TNBC) subtype, this regulatory network demonstrates distinct heterogeneity and drug resistance characteristics. The core role of ZEB1 is further amplified: it forms a positive feedback loop with ZCCHC24, maintaining stem cell properties [138, 199], and breast cancer susceptibility gene 1/2-containing complex subunit 3 (BRCC3) stabilizes it through deubiquitination [200]. Metabolically, the function of ZFP64 expands: it is upregulated in response to histone lactylation induced by lactate in the tumor microenvironment. This subsequently inhibits ferroptosis and contributes to chemotherapy resistance through activation of GTP cyclohydrolase-1 (GCH1) and ferritin heavy chain 1 (FTH1) [71]. Regarding immune and therapeutic resistance, loss of ZFP652 leads to upregulation of PD-L1 and T cell exhaustion [99], whereas silencing of ZFP689 activates long interspersed element-1 (LINE-1) retrotransposons, exacerbating tumor heterogeneity and impairing immunotherapy responses [101].

In summary, the functions of ZFPs in breast cancer are not fixed, but exhibit considerable diversity and context dependency. ZEB1 serves as a representative example: it acts as a core driver of EMT and metabolic reprogramming in pan–breast cancer, while its role is further amplified in TNBC. This amplification is reflected not only through reinforcement by a positive feedback loop with ZCCHC24 [138] and stabilization via BRCC3-mediated deubiquitination [200], but also through its critical extension into the regulation of ferroptosis sensitivity [44], thereby directly influencing TNBC survival and metastatic potential. ZFP64 further illustrates how a single ZFP can exert oncogenic effects through distinct downstream pathways across subtypes. It drives the Warburg effect in conventional breast cancer [70], and in TNBC, it serves as a key molecular bridge connecting microenvironmental signals to intrinsic ferroptosis resistance [71]. This functional duality highlights the precision and complexity of the ZFP regulatory network, presenting both challenges and opportunities for developing precision medicine strategies tailored to specific molecular backgrounds.

#### Colorectal cancer

Colorectal cancer (CRC) ranks as the third most common malignant tumor worldwide [192], and its initiation and progression constitute a complex multistep process involving numerous genes and signaling pathways. ZFPs form an extensive molecular network that drives malignant progression through precise regulation of key biological events, including cell proliferation, apoptosis, EMT, and metabolic reprogramming.

During tumor initiation and growth, ZFPs promote proliferation and suppress apoptosis by modulating core signaling pathways. For example, ZFP695 accelerates the cell cycle and inhibits apoptosis via activation of the PI3K/Akt/mTOR pathway [102]. Similarly, ZFP280A utilizes this pathway to promote tumor growth by modulating ribosomal protein stability [83]. Within the Wnt/β-catenin pathway, SALL2/ZFP795 exerts tumor-suppressive effects by upregulating the negative regulator axis inhibitor 2 (AXIN2), thereby inhibiting β-catenin nuclear translocation [105]. ZFP277, identified as a target of this pathway, induces p21WAF1-mediated senescence upon its loss, collectively contributing to tumor suppression [82].

As tumors progress to invasive and metastatic stages, EMT emerges as a central biological process. ZEB1, a core regulator of EMT, has its activity precisely modulated by upstream factors, such as ribosomal RNA processing 12 homolog (RRP12) and Timeless [201, 202]. It subsequently promotes tumor growth by recruiting the NuRD complex to transcriptionally inhibit glycolysis-related tumor suppressor genes [45]. Its activity is further negatively regulated by myosin heavy chain 11 (MYH11) [203]. Meanwhile, other ZFPs influence invasive capacity through regulation of matrix-degrading enzymes. ZFP24 directly suppresses matrix metallopeptidase 2 (MMP2) transcription [67]. Conversely, ZFP384 enhances MMP2 expression under hypoxia inducible factor 1 subunit alpha (HIF-1α) induction, illustrating the regulatory complexity within this protein family [93].

Epigenetic mechanisms significantly influence ZFP function in CRC. Multiple ZFPs, including ZFP334, ZFP671, and ZBTB18, act as tumor suppressor genes. However, their activity is frequently silenced by promoter hypermethylation [60, 204, 205]. Notably, ZEB1’s promoter methylation status itself determines its functional propensity: hypomethylation promotes invasion, while hypermethylation correlates with a favorable prognosis [206]. Beyond transcriptional regulation, ZFPs also operate through non-canonical mechanisms. For instance, MORC2 promotes tumor progression and EMT by regulating alternative splicing of CDK5RAP2 [134], while ZFP36L1/L2 inhibites proliferation by degrading G1/S-specific cyclin-D1 (CCND1) mRNA [127]. This broadens the understanding of ZFP functional diversity.

Within the tumor microenvironment, ZFPs serve as central mediators, bridging intracellular and extracellular signaling. In inflammatory contexts, ZEB1 indirectly promotes metastasis by suppressing inflammatory responses in cancer-associated fibroblasts [207]. Importantly, gut microbiota accelerates tumor progression by triggering ZFP90 expression via the toll-like receptor 4 (TLR4)–PI3K–AKT–NF-κB axis. This establishes a molecular link between microbial presence and tumor evolution [73].

In summary, ZFPs constitute a multi-layered regulated network in colorectal cancer. Future research should focus on several key directions. First, systematically elucidating the genome-wide binding profiles and co-regulatory networks of pivotal ZFPs, such as ZEB1, across different microenvironments is essential. This may reveal molecular switches underlying their functional plasticity. Second, deepening the exploration of ZFPs’ bidirectional roles within the tumor microenvironment is also crucial, particularly in mediating microbiota–tumor crosstalk, as exemplified by ZFP90. These investigations will provide critical insights for the rational design of combination therapy strategies.

#### Hepatocellular carcinoma

Hepatocellular carcinoma (HCC) represents the third leading cause of cancer-related deaths globally [192]. Its high mortality rate is closely associated with robust proliferative capacity, invasive potential, and inherent treatment resistance. Within this complex molecular landscape, the ZFP family orchestrates several core biological processes, including cell cycle regulation, metabolic reprogramming, EMT, and therapy resistance. Thus, ZFPs form a multi-layered regulatory network that drives HCC initiation and malignant progression.

In the context of cell cycle and proliferation control, ZFPs disrupt normal checkpoint regulation through diverse molecular mechanisms. For instance, MAZ promotes transcriptional condensate formation at the CCND1 promoter via aberrant liquid–liquid phase separation. This directly drives cyclin D1 overexpression and tumor proliferation [106]. Furthermore, ZFP131 upregulates poly(A) binding protein interacting protein 1 (PAIP1) through the yes-associated protein 1 (YAP1) signaling pathway to activate AKT. It also directly activates structural maintenance of chromosomes protein 4 (SMC4) expression, thereby coordinating cell cycle progression via dual regulatory pathways [75, 76].

ZFPs also constitute a precise regulatory network governing EMT and metastatic. MAZ promotes ZEB1 m⁶A methylation by transcriptionally regulating methyltransferase-like protein 3 (METTL3), thereby facilitating EMT [208]. Zinc finger MYM-type containing 1 (ZMYM1), itself a target of METTL3-mediated m⁶A modification, synergistically regulates E-cadherin downregulation and N-cadherin upregulation through activation of the RAS/ERK/c-FOS pathway. This collectively enhances HCC metastasis [139].

Metabolic reprogramming represents another critical dimension of ZFP function, supporting HCC malignant progression and establishing feedback loops with the tumor microenvironment. ZEB1 exerts multi-layered metabolic control. It directly binds the phosphoglycerate dehydrogenase (PHGDH) promoter to activate the serine synthesis pathway [46] and also simultaneously activates phosphofructokinase-1 (PFKM) transcription to enhance glycolysis [209]. ZFP207 promotes aerobic glycolysis through transcriptional regulation of enolase 1 (ENO1) and glyceraldehyde-3-phosphate dehydrogenase (GAPDH) [79]. Notably, ZFPs also link metabolic alterations to immune modulation. ZMYND8 recruits bromodomain containing 4 (BRD4) to the hexokinase 2 (HK2) promoter to enhance glycolysis [142]. Furthermore, ZFP296 modulates the tumor immune microenvironment via interactions with immune cells [210].

Treatment resistance in HCC is closely tied to the adaptability of these molecular networks. Beyond its roles in EMT and metabolism, ZEB1 mediates sorafenib resistance by promoting mitochondrial fission [211]. At the epigenetic level, MAZ recruits proteins such as histone deacetylase 1 (HDAC1) to silence the tumor suppressor gene C-Src tyrosine kinase (CSK). This leads to activation of multiple oncogenic signaling pathways [212]. Additionally, ZFP498 established an epigenetic basis for treatment resistance through direct interaction with p53, thereby suppressing p53-mediated apoptosis and ferroptosis [97]. Notably, upregulated ZFP64 is phosphorylated by protein kinase C alpha (PKCα). This leads to its nuclear translocation and subsequent activation of macrophage colony-stimulating factor 1 (CSF1), which polarizes macrophages toward an M2 phenotype, thereby driving immune evasion and anti-PD1 resistance [72].

In summary, ZFPs form a multifunctional and highly interconnected regulatory network in HCC. Key node proteins, such as MAZ and ZEB1, exhibit remarkable functional diversity. MAZ drives cell cycle progression via phase separation [106], influences EMT [208], and contributes to treatment resistance [212] through epigenetic mechanisms. Concurrently, ZEB1 coordinates EMT [208], metabolic reprogramming [46, 209], and drug resistance [211]. This functional versatility enables a limited set of ZFPs to act as molecular hubs, synchronously regulating the evolution of multiple malignant phenotypes. Future research aimed at systematically dissecting this intricate regulatory circuitry will offer a solid theoretical foundation for developing rational combination therapies targeting key nodes within the ZFP network.

**Fig. 3:**
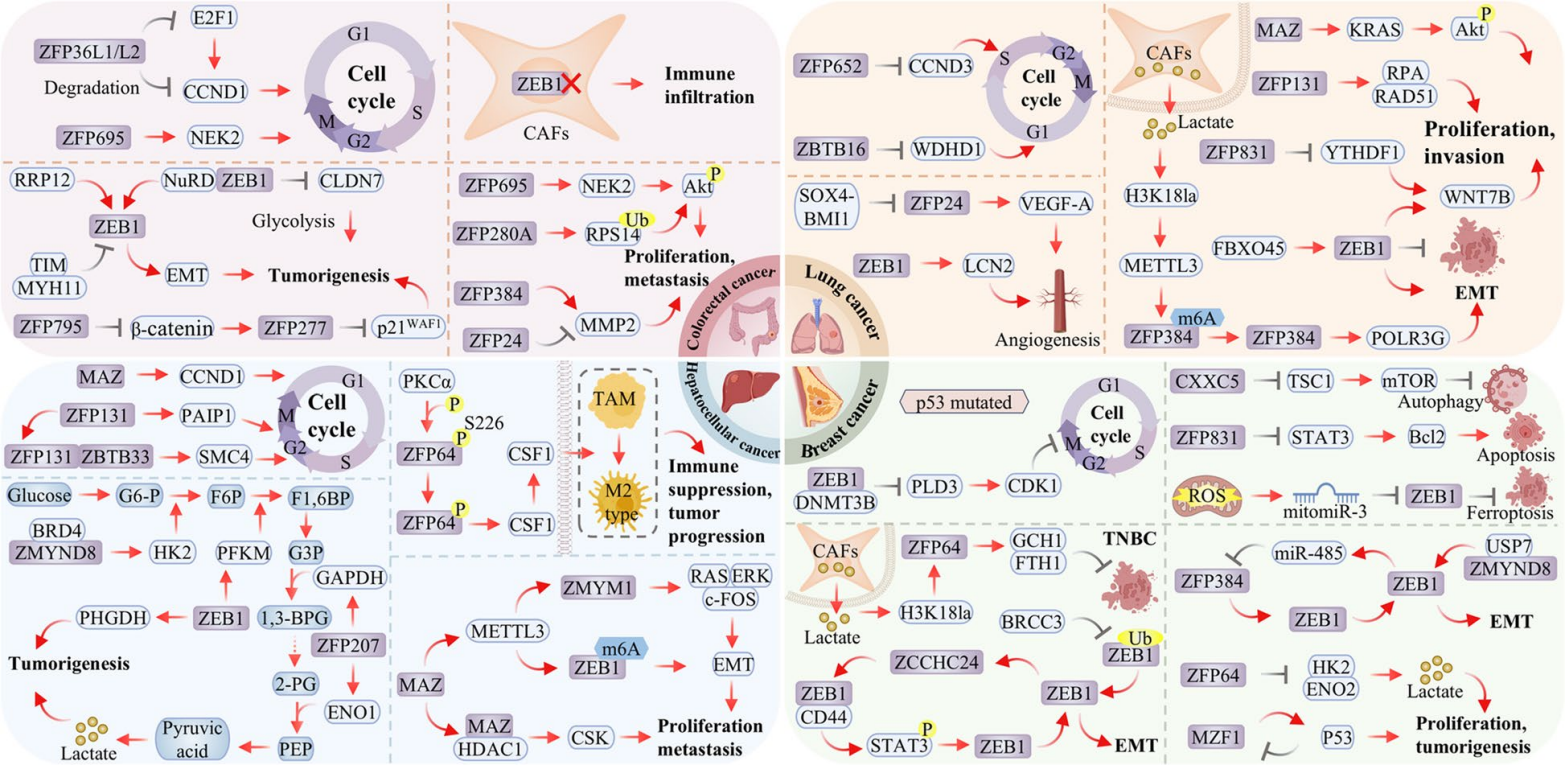
Role of ZFPs in cancer. ZFPs constitute pivotal molecular regulators across four major cancer categories: lung cancer, hepatocellular cancer, breast cancer, and colorectal cancer, ZFPs critically govern tumorigenesis by modulating cell cycle progression, proliferation, and metastatic migration. Abbreviations: (CAFs: cancer-associated fibroblasts; TAMs: tumor-associated macrophages; TNBC: triple-negative breast cancer; EMT: epithelial-mesenchymal transition; SSP: serine synthesis pathway; G6-P: Glucose-6-Phosphate; HK2: Hexokinase 2; F6P: Fructose-6-Phosphate; F1,6BP: Fructose-1,6-bisphosphate; G3P: Glyceraldehyde-3-Phosphate; 1,3-BPG: 1,3-Bisphosphoglycerate; 2-PG: 2-Phosphoglycerate; PEP: Phosphoenolpyruvate; PFKM: Phosphofructokinase, Muscle type; GAPDH: Glyceraldehyde-3-Phosphate Dehydrogenase; ENO1: Enolase 1)

In conclusion, in multiple cancer types, ZFPs have been established as central hubs that coordinate fundamental processes including proliferation, metabolic reprogramming, EMT, and immune evasion to drive progression (Fig. 3). The high context-dependency of ZFPs, shaped by cell-type-specific signals and microenvironmental pressures, underlies tumor adaptation and therapy resistance. Thus, not simply oncogenic or tumor-suppressive are ZFPs; rather, they function as plastic, context-dependent regulators whose physiological roles can be hijacked or inactivated in tumors. Therefore, required for effective targeting are strategies that precisely modulate their context-specific activity, moving beyond broad inhibition or activation. Future efforts should be directed toward defining the dynamic regulatory maps and molecular switches that control ZFP functions, thereby enabling the design of context-aware combination therapies.

### Central nervous system disorders

CNS disorders encompass a spectrum of complex pathological states ranging from acute injuries to chronic degenerative conditions. ZFPs are deeply implicated in their pathological processes by forming disease-specific regulatory networks. In the cascading pathological processes of stroke, predominantly ischemic stroke (IS), ZFPs are involved in multiple facets spanning risk factors, acute injury, tissue repair and blood–brain barrier disruption. In neurodegenerative diseases such as AD and PD, they primarily regulate core processes including abnormal protein aggregation and clearance as well as mitochondrial dysfunction; additionally, in amyotrophic lateral sclerosis (ALS), ZFPs play a pivotal role in aberrant RNA metabolism and protein homeostasis imbalance. In epilepsy and depression, by contrast, ZFPs exert prominent effects on the balance of neuronal excitability and neurotransmitter systems. This functional diversity underscores the extensive and critical role of ZFPs in the pathology of the CNS.

#### Stroke

CNS disorders represent a spectrum of severe conditions that range from acute injuries to chronic degenerative pathologies. Among these, stroke, an acute neurological condition, primarily results from the sudden rupture or occlusion of cerebral blood vessels, leading to disrupted blood supply. It represents one of the leading global causes of mortality and long-term disability. Epidemiological studies indicate a disproportionate burden in low- and middle-income countries, which constitute 86% of global stroke cases [213]. Pathogenically, stroke is classified into two major types: ischemic and hemorrhagic. Epidemiological data from 2019 demonstrate that IS accounts for 62.4% of all stroke cases worldwide, highlighting its significant public health burden [214]. With demographic aging and evolving lifestyle factors, the annual global incidence of ischemic stroke is projected to reach 4.9 million by 2030 [215]. The condition causes over 70 million healthy life-years lost annually, with ischemic stroke responsible for approximately 43% of all stroke-related disability-adjusted life-years (DALYs) [216]. Despite advancements in therapeutic approaches, current standard treatments for ischemic stroke, intravenous thrombolysis and endovascular therapy, are limited by a narrow therapeutic time window, restricting their applicability to only a portion of eligible patients [217]. Furthermore, thrombolytic treatments carry significant risks, including symptomatic intracranial hemorrhage, with safety profiles varying among agents such as alteplase and tenecteplase [218]. Research has extensively documented the involvement of ZFPs across the entire continuum of ischemic stroke, from risk factor modulation to its multifaceted pathophysiological mechanisms. Within ischemic brain injury, ZFPs frequently exhibit context-dependent, dual regulatory roles. Their net effect is determined by the timing of expression, cellular microenvironment, and interactions with upstream and downstream signaling pathways. The subsequent discussion will focus on these complex roles of ZFPs in ischemic stroke.

Before exploring the role of ZFPs in the core pathological mechanisms of ischemic stroke, it is necessary to understand their involvement in the pathogenesis of its major risk factors. ZFPs have been extensively implicated in the pathogenesis of major risk factors for IS, including hypertension, hyperglycemia, and atherosclerosis. Their functions span systemic regulatory pathways and local vascular effects, offering a molecular perspective on how these risk factors contribute to stroke susceptibility.

Hypertension is a key modifiable risk factor that alters cerebral hemodynamics and increases stroke severity [219]. ZFPs act as critical integrators of neuroendocrine and vascular pathways in hypertension. The mineralocorticoid axis is a central hormonal driver [220]. Within this axis, CASZ1 (ZFP693) serves as a fundamental inhibitory regulator. Its impairment leads to overactivation of the aldosterone–MR pathway, promoting volume and pressure overload [221]. At the vascular level, ZFP36 enhances vascular tone by destabilizing G protein signaling 2 (RGS2) mRNA, thereby sustaining vasoconstriction [128]. Kruppel-like factor 5 (KLF5), upregulated by complement C3a via ERK, promotes a pathological synthetic phenotype in VSMCs. It also stimulates renin secretion via liver X receptor alpha (LXRα), thereby reinforcing the renin-angiotensin system [34]. Interventionally, suppression of ZFP36 via AAV9-shRNA normalizes blood pressure in hypertensive models [128]. Thus, ZFPs operate within a multi-tiered regulatory system, linking systemic neuroendocrine control to local vascular tone.

Hyperglycemia exacerbates stroke risk through metabolic dysregulation and vascular injury [222]. ZFPs coordinate systemic glucose homeostasis by integrating central sensing and peripheral tissue functions. Juxtaposed with another zinc finger gene 1 (JAZF1) enhances systemic insulin sensitivity via hypothalamic InsR-PI3K-Akt signaling [108]. The Krüppel-like zinc finger transcription factor Gli-similar 3 (GLIS3) maintains β-cell function by transactivating insulin gene (Ins2) and Mafa [118]. In diabetes, miR-200c overexpression targets JAZF1, contributing to β‑cell apoptosis [223]. Vascular damage under diabetic conditions involves KLF5. Its reduction in diabetic wounds impairs C-X-C motif chemokine 12 (CXCL12) -mediated neovascularization [35]. High glucose also activates KLF5 in endothelial cells, disrupting angiogenesis [224]. Furthermore, the ZEB1–miR-200 feedback loop, which normally preserves β-cell identity, is disrupted in diabetes. This promotes β-cell exhaustion and pro-inflammatory VSMC switching [225]. These mechanisms collectively link hyperglycemia-driven microvascular dysfunction to impaired cerebral autoregulation and lacunar infarction [226].

Atherosclerosis, as a critical underlying pathology, involves ZFPs in context-dependent roles encompassing endothelial dysfunction, inflammation, and plaque stability. Hemodynamic forces regulate KLF2/4 expression in endothelial cells. Stable flow upregulates these ZFPs to suppress inflammation and thrombosis. Conversely, disturbed flow downregulates them, promoting a pro-atherogenic phenotype [227]. KLF4 exhibits cell-type-specific duality. In macrophages, its restoration promotes M2 polarization and plaque stabilization [228] However, in VSMCs, KLF4 drives phenotypic switching and inflammation [29]. In endothelial cells, KLF4 inhibits atherosclerosis via endonuclease/exonuclease/phosphatase family domain‐containing 1 (EEPD1) suppression [229]. Furthermore, KLF2/4 function as key regulators of thrombosis. They induce thrombomodulin and endothelial nitric oxide synthase (eNOS) while suppressing plasminogen activator inhibitor-1 (PAI-1) expression, ultimately prolonging clotting time [26]. Similarly, ZEB1 promotes plaque stability in myeloid cells [230], yet in endothelial cells, it upregulates vascular cell adhesion molecule 1 (VCAM1)/intercellular cell adhesion molecule 1 (ICAM1) to enhance inflammation [49]. ZEB2 regulates VSMC phenotype through chromatin remodeling. Its deletion leads to high-risk lesions [54]. Neointimal hyperplasia is modulated by several ZFPs. ZFP36 exerts an inhibitory effect [129], Kruppel-like factor 13 (KLF13) promotes dedifferentiation via smooth muscle protein 22 α (SM22α) [41], GATA zinc finger transcription factor family 6 (GATA6) regulates proliferation and migration [146]. Phosphorylated GATA2 accelerates plaque formation via VSMC transdifferentiation [144].

Collectively, ZFPs function as molecular hubs within the ischemic stroke risk network. They not only independently drive each risk factor but also produce synergistic effects through shared regulatory nodes, such as KLF and ZEB family members. This amplifies overall stroke risk. For example, KLF5 activation in hypertension and its dysregulation under hyperglycemic conditions can synergistically worsen vascular remodeling and dysfunction. Similarly, the chronic inflammatory state common to both hypertension and diabetes can exacerbate dysregulated inflammatory signaling in atherosclerosis. Notably, the context-dependent functions of ZFPs, exemplified by KLF4 and ZEB1, offer important insights into stroke risk mechanisms. These apparent functional contradictions are not coincidental; instead, they reflect divergent cellular responses to the same risk signal during the prolonged preclinical phase of stroke. For instance, KLF4 promotes plaque stability in macrophages yet drives a destabilizing phenotypic switch in VSMCs [29, 228]. Likewise, ZEB1 enhances plaque stability in myeloid cells but aggravates inflammatory responses in endothelial cells [49, 230]. This indicates that systemic upregulation or inhibition of a given ZFP may be ineffective or even harmful for stroke prevention. Instead, the net effect depends on its cell-type-specific functional balance within stroke-vulnerable tissues, such as different cellular components of unstable plaques and diabetic cerebral microvessels. This cell- and context-specificity not only explains the individual variability in stroke risk but also points toward future cell-type-targeted therapeutic strategies. Precision modulation of ZFP activity within specific pathological cell populations could allow safer and more effective intervention at the source of multiple risk factors, thereby reducing the incidence of ischemic stroke. Ultimately, ZFPs form the pathogenic basis of stroke by regulating multiple risk factors. Once a stroke occurs, these proteins also play a central role in the pathophysiological cascade during the acute phase.

Following the identification of various risk factors for ischemic stroke, understanding how these factors specifically drive disease onset and progression is essential. The pathology of ischemic stroke unfolds as a dynamic, multi-stage cascade. Its core initiation involves acute cerebral vessel occlusion or stenosis, leading to cerebral ischemia and hypoxia. This initial event immediately triggers a series of intricate and interconnected pathophysiological responses. Oxidative stress arises first, subsequently triggering a robust inflammatory reaction. Together, these processes amplify cellular damage and activate programmed cell death pathways, including apoptosis, necroptosis, and ferroptosis. Concurrently, ischemia and subsequent reperfusion directly affect the cerebral vasculature, initiating a complex process of vascular injury and repair. However, the body’s repair mechanisms often become unbalanced or insufficient. This repair process, characterized by inflammatory cell infiltration and matrix metalloproteinase release, along with persistent ischemic injury, further compromises the structure and function of the BBB. The resulting increase in BBB permeability leads to vasogenic edema and allows toxic substances to enter the brain, thereby markedly amplifying the initial ischemic injury and establishing a vicious cycle.

The core pathological mechanism of ischemic stroke originates from the cascading damage effects induced by hypoxia–ischemia, which disrupt intracellular redox balance and metal ion homeostasis, thereby triggering a vicious cycle of neuronal injury [231]. During these process, abnormal release of Zn^2^⁺ from presynaptic neurons occurs, leading to a sharp increase in cytoplasmic zinc concentration and establishing pathological zinc homeostasis imbalance [232]. This zinc dysregulation directly activates Metallothionein (MT) gene expression, with its regulatory mechanism being partially dependent on elevated intracellular zinc levels [233]. Notably, MT serves as a dynamic zinc reservoir that reversibly releases zinc ions under oxidative stress conditions, subsequently activating metal regulatory transcription factor 1 (MTF-1) [234]. Activated MTF-1 translocated into the nucleus and recruited transcription factors such as hypoxia-inducible factor-1α (HIF-1α) to the promoter region of the *MT* gene. This process regulates the induction of *MT* gene expression in response to hypoxia [235]. Collectively, this pathway constitutes a complete signaling axis from zinc homeostasis imbalance to MT activation and MTF-1-mediated transcriptional regulation. Within 2 h following ischemia, MTI and MTII are swiftly upregulated, with their expression levels peaking at 16 h [236], and post-ischemic upregulation of MTIII was found to contribute significantly to neuroprotection by chelating synaptic Zn^2^⁺ and mitigating zinc-mediated excitotoxicity [237]. After MTII-deficient mice were subjected to middle cerebral artery occlusion (MCAO), a marked elevation of hippocampal 8-OHdG levels was detected. Moreover, in vitro experiments have demonstrated that MTIII is capable of scavenging superoxide anions [153]. Furthermore, zinc ion promoted iron chelation, which in turn leads to a reduction in the iron concentration within lysosomes after oxidative stress [238].Besides, exogenous administration of MTII resulted in a significant reduction in both direct and indirect infarct volumes, along with an improvement in neurological deficits [239]. These collective findings strongly suggest that MT might be an effective target against ischemic stroke.

Beyond its direct regulation of MT, MTF-1 also cooperates with SP1 to coordinate ion homeostasis and promote cell survival. Specifically, it directly transactivates the sodium-calcium exchanger 1 (NCX1) gene by binding to the metal response element (MRE) within the promoter region. This interaction facilitated the regulation of intracellular Na⁺/Ca^2^⁺ homeostasis [121]. Under ischemic conditions, ROS upregulate SP1 protein levels through IRES-dependent translational activation, which serves as an endogenous protective mechanism [240].SP1 interacts with hypoxia-inducible factor 1 (HIF-1) to bind to the brain promoter region of NCX1 (ncx1-Br), recruiting histone acetyltransferase p300 and inducing hyperacetylation of histones in this region. This hyperacetylated state promoted the transcription of NCX1, leading to its upregulation [120]. Furthermore, SP1-mediated upregulation of TP53-induced glycolysis and apoptosis regulator (TIGAR) following ischemia/reperfusion enhances NADPH and reduces glutathione production via the pentose phosphate pathway, thereby facilitating ROS clearance [119]. Given the integration of zinc signaling, antioxidant defense, and ion homeostasis pathways in neurological disorders through the "MTF-1-MT-SP1" axis, targeting this axis therefore represents a promising therapeutic strategy.

Beyond the established MT-MTF-1 axis, additional ZFPs participate in the oxidative stress response. Ischemic preconditioning significantly upregulated ZFP667 expression in the hippocampus and cortex. ZFP667 overexpression subsequently reduced lactate dehydrogenase (LDH) release and increased cell viability, demonstrating its direct role in mitigating oxidative stress-induced cell death [100]. Concurrently, glioma-associated oncogene homolog 1 (Gli1) was identified as a key downstream transcription factor of the Sonic Hedgehog pathway activated under ischemia. This activation initiated transcription of the superoxide dismutase 1 (SOD1) gene, thereby enhancing ROS clearance capacity [122]. In summary, ZFPs coordinate a multi-layered defense against oxidative stress through both the canonical MT-MTF-1 axis and alternative pathways involving ZFP667 and Gli1. Their ability to integrate zinc homeostasis, antioxidant gene regulation, and metabolic adaptation positions them as central players in mitigating the initial oxidative insult of cerebral ischemia.

Following oxidative stress, the secondary neuroinflammatory response triggered by ischemia serves as a critical link leading to brain damage and functional deficits. This involves the activation and phenotypic polarization of brain-resident immune cells, primarily microglia and astrocytes. ZFPs play a pivotal role in orchestrating this process by modulating glial polarization and fine-tuning key inflammatory signaling pathways. They thus shape the post-ischemic neuroinflammatory landscape.

Multiple studies have established KLF4 as a critical regulator of glial polarization. In astrocytes, KLF4 is induced following ischemic injury. It inhibits A1 astrocyte activation while promoting A2 polarization after oxygen–glucose deprivation/reoxygenation through NF-κB regulation [241]. In microglia under ischemic conditions, KLF4 was found to directly bind to the promoter region of Arg1, inducing its transcription and promoting M2-type polarization of microglia [242]. Furthermore, the acetylation status of KLF4 can induce the phenotypic transition of microglia from M1 to M2, which similarly alleviates brain injury and reduces inflammatory responses [243]. Conversely, another study revealed that the upregulation of long noncoding RNA (lncRNA) maternally expressed gene 3 (MEG3) could suppress the expression of KLF4, leading to the polarization of microglia towards the M1 phenotype and thereby exacerbating neuroinflammation and brain injury [244]. These findings collectively suggest that restoring KLF4 activity may contribute to alleviating neuroinflammatory responses and brain injury induced by ischemic stroke.

TLR4 has been identified as a core initiator of neuroinflammation in stroke. Notably, excessive TLR4 activation has been shown to significantly suppress the expression of the MCPIP1 (ZC3H12A). MCPIP1, a newly recognized mRNA endonuclease, exerts negative feedback regulation by specifically recognizing and effectively degrading mRNAs that encode pro-inflammatory cytokines. Besides, MCPIP1 functions as a deubiquitinating enzyme, negatively regulating the JNK and NF-κB signaling pathways by targeting tumor necrosis factor receptor-associated factors (TRAFs) [147]. Electroacupuncture (EA) pretreatment significantly upregulated the protein and mRNA expression levels of MCPIP1 in the mouse brain. Further studies demonstrated that in wild-type mice, EA pretreatment significantly reduced the expression levels of pro-inflammatory cytokines in brain tissue after MCAO and markedly inhibited activation of the NF-κB signaling pathway post-MCAO. However, in MCPIP1-deficient mice, EA pretreatment failed to exert these inhibitory effects [245]. Similarly, A20(TNFAIP3), functioning as a negative regulator of the NF-κB pathway, could reduce the production of inflammatory factors and promote the polarization of microglia towards the M2 phenotype [150].

Furthermore, in experimentally induced ischemic stroke models, the upregulation of ZEB1 within microglia has been identified as a central regulatory mechanism. ZEB1 directly promoted the transcriptional activation of transforming growth factor beta 1 (TGF-β1) and inhibited the secretion of CXCL1 by astrocytes, thereby alleviating inflammatory responses through decreases neutrophil infiltration [50]. Besides, ZEB1 binds to the promoter region of G protein-coupled receptor 30 (GPR30) and enhances its transcriptional activity. GPR30, functioning as an estrogen receptor, inhibits the TLR4/NF-κB signaling cascade when activated, thereby alleviating inflammatory responses and brain tissue damage [246]. Collectively, these findings establish ZFPs as master regulators of post-ischemic neuroinflammation. They fine-tune glial polarization and cytokine signaling through diverse mechanisms. Their coordinated actions shape the inflammatory landscape after stroke, offering multiple nodal points for therapeutic intervention.

In the pathological cascade of ischemic stroke, programmed cell death represents a central mechanism responsible for the delayed loss of neurons. As critical transcriptional regulators, ZFPs participate extensively in and integrate multiple cell death pathways, including apoptosis, necroptosis, and ferroptosis, by modulating their core molecular components, thereby shaping the final outcome of neural injury.

Research has demonstrated that members of the KLF family play central roles in regulating post-ischemic apoptosis, with distinct functional outcomes depending on the specific member. KLF2, KLF4, and KLF11 exerte anti-apoptotic effects. Specifically, KLF2 reduced microglial apoptosis by decreasing the Bax/Bcl-2 ratio and suppressing caspase-3/9 expression [27]. KLF4 alleviated mitochondrial damage by regulating obesity-associated protein (FTO) via lncRNA zinc finger antisense 1 (ZFAS1) [31] and counteracted oxygen–glucose deprivation-induced pro-apoptotic factor expression through upregulation of metastasis associated lung adenocarcinoma transcript 1 (MALAT1) [247]. KLF11 formed a complex with peroxisome proliferator-activated receptor gamma (PPARγ) to cooperatively inhibit the transcription of pro-apoptotic microRNA-15 (miR-15) [39]. Conversely, Kruppel-like factor 9 (KLF9) promoted injury; its inhibition, however, enhanced neuronal survival by upregulating TNF receptor-associated factor 2 (TRAF2) -mediated KLF2 [248].

The zinc finger protein A20 functions as a regulatory hub in both apoptosis and necroptosis. Through its deubiquitinating activity, A20 fine-tunes K63-linked ubiquitination of receptor-interacting serine/threonine-protein kinase 1 (RIPK1), thereby influencing the formation of the RIPK1/caspase-8 complex and the initiation of apoptosis [151, 249]. In vitro experiments utilizing Nucleofector electroporation transfection demonstrated that A20-transfected primary hippocampal neurons exhibit significant resistance to TNF-α-induced apoptosis. In vivo, transplantation of A20-overexpressing neurons into the ischemic penumbra of MCAO-treated rats revealed markedly higher survival rates of A20-overexpressing neurons compared to controls at 3 and 7 days post-transplantation [250]. In necroptosis pathways, A20 protein levels declined after ischemia, accompanied by upregulation of key mediators such as receptor-interacting protein kinase (RIPK3) and mixed lineage kinase domain-like protein (MLKL)/p-MLKL, as well as enhanced K63-linked ubiquitination of RIPK3 [152]. Qiu et al. demonstrated that hypoxic preconditioning (HPC) could upregulate A20, thereby inhibiting RIPK3-dependent necroptosis and the M1 polarization of microglia/macrophages induced by necroptosis [251].

ZFPs also play critical roles in regulating ferroptosis. Studies demonstrated that KLF6, by suppressing the activation of the nuclear factor erythroid 2-related factor (Nrf2)/heme oxygenase 1 (HO-1) signaling pathway, indirectly resulted in the accumulation of lipid peroxides. It also upregulated the expression of pro-ferroptotic genes (e.g., ACSL4) while downregulating the expression of anti-ferroptotic genes (e.g., GPX4), ultimately promoting ferroptosis [37]. The transcription factor SP1 exhibits context-dependent effects. In neurons, SP1 bound to and promoted the transcription of ACSL4 [252]. While it also bound to the GPX4 promoter, ischemic conditions led to histone deacetylase 9 (HDAC9) -mediated degradation of SP1, which reduced GPX4 transcription and indirectly promoted ferroptosis [253]. Conversely, in brain microvascular endothelial cells, SP1 functions as the primary transcription factor for tumor necrosis factor superfamily member 9 (TNFSF9), driving ferroptosis by upregulating TNFSF9 to suppress solute carrier family 3 member 2 (SLC3A2) expression [254].

Notably, certain ZFPs display functional duality or strong context-dependency. For instance, the role of ZEB1 in ischemic brain injury is not uniform. In permanent cerebral ischemia models, ZEB1 expression was transcriptionally upregulated by P63, leading to the blockade of neuronal apoptosis pathways through the repression of pro-apoptotic targets such as transactivation-competent p73 (TAp73) [255]. Conversely, within the forkhead box O 3a (FOXO3a)/SPROUTY2 (SPRY2)/ZEB1 neuroprotective signaling axis, modulated by Methyl CpG binding protein 2 (MeCP2) overexpression in ischemic stroke, ZEB1 expression was suppressed. This suppression was beneficial for neuroprotection [256]. These seemingly contradictory findings indicate that the net effect of ZEB1 depends heavily on specific spatiotemporal contexts and molecular microenvironments. Acute induction may represent a protective response, whereas sustained overexpression might exert detrimental effects through distinct downstream targets.

Collectively, ZFPs form a sophisticated and precise regulatory network that orchestrates the activation and balance of multiple programmed cell death pathways after cerebral ischemia. Key members, including the KLF family, A20, ZEB1, and SP1, act as central decision-makers across different death modalities. Their widespread functional duality and high context-dependency underscore the complexity of ischemic brain injury and suggest that future neuroprotective strategies targeting these molecules must achieve enhanced cell-type specificity and spatiotemporal precision.

In addition to determining cell survival or death, the vascular response and repair capacity after ischemia directly influence the restoration of cerebral blood flow and long-term functional outcomes in brain tissue. A major obstacle in ischemic stroke recovery is the “no-reflow” phenomenon, where microcirculatory reperfusion fails despite macrovascular recanalization. This complication affects approximately 25% of patients and severely limits clinical outcomes [257]. Consequently, the activation of endogenous angiogenesis becomes critical for restoring microvascular perfusion and promoting long-term neurological function. VEGF, as a primary angiogenic effector, promotes endothelial proliferation and migration, thereby driving neovascularization [258]. In contrast, a study found that after ischemic stroke, ZFP24, as a Runx1 co-regulator, suppresses VEGFA transcription/expression, impairing angiogenesis [259]. Moreover, the knockdown of MCPIP1 was found to inhibit the expression of VEGF and HIF-1α [260]. However, in ischemic stroke, the knockdown of MCPIP1 could activate the AKT/mTOR signaling axis by upregulating the transferrin receptor (TFRC). This, in turn, increased the expression of VEGF, angiopoietin-1, and HIF-1α, thereby promoting the proliferation, migration, and angiogenic capacity of endothelial colony-forming cells (ECFCs) both in vitro and in vivo [148]. Additionally, KLF4 has been shown to exert multiple protective vascular effects, including indirect inhibition of cell adhesion molecules (CAMs) expression through suppression of NF-κB phosphorylation, enhancement of VSMC contractility and stability, and promotion of endothelial cell proliferation and migration via Wnt/β-catenin activation [32, 261].

Interleukin-6 (IL-6) has been established as a pro-inflammatory cytokine, and its elevation has been associated with a worse stroke outcome. However, paracrine IL-6 has been implicated in stroke recovery. In stroke models, the knockout of ZFP580 was found to reduce paracrine IL-6 but increase soluble form of the Il6 receptor (sIl6r) levels, thereby promoting IL-6 trans-signaling. In this process, IL-6 can bind to sIL-6R and activate the glycoprotein 130 (gp130) receptor, which facilitates angiogenesis and the repair process [262]. Moreover, the knockout of ZFP580 increased the levels of endocrine insulin-like growth factor 1 (Igf1) in the blood and elevated the Igf1/insulin-like growth factor binding protein 3 (Igfbp3) molar ratio. As a result, more unbound, active Igf1 could cross the blood–brain barrier and enter the brain, exerting its effects on angiogenesis and repair promotion, thereby comprehensively ameliorating stroke outcomes [263]. These findings collectively demonstrate that ZFPs regulate multiple facets of vascular responses to ischemia, from initial angiogenic stimulation to larger vessel remodeling, highlighting their therapeutic potential for promoting vascular recovery.

In ischemic injury, the disruption of the vascular system directly manifests as the breakdown of BBB integrity. This BBB breakdown represents a critical pathological event that amplifies secondary injury by permitting uncontrolled entry of neurotoxic substances into the brain parenchyma, thereby exacerbating vasogenic edema, hemorrhagic transformation, and inflammatory cell infiltration [264]. Occludin, a transmembrane protein localized within endothelial plasma membranes, serves as a core component of tight junctions (TJs). Its degradation has been confirmed as a critical determinant of BBB disruption, thereby accelerating the pathology of ischemic stroke [265]. Previous in vitro and in vivo studies revealed that KLF2 cloud protect the BBB by regulating the tight junction factor occludin [28]. Subsequently, it has been demonstrated that phoenixin-14 and Netrin-1 cloud confer protective effects on BBB integrity following I/R injury by activating the KLF2/occludin signaling pathway [266, 267]. Furthermore, KLF4 was similarly shown to upregulate the Nrf2/Trx1 signaling pathway to elevate levels of TJs [30]. Complementing these findings, KLF11 was found to directly bind the promoter regions of occludin and zonula occludens-1 (ZO-1), enhancing their transcriptional activity [40]. In contrast to these protective KLF members, MCPIP1 deficiency induced severe barrier disruption through matrix metalloproteinase (MMP) upregulation and tight junction protein reduction [149]. Thus, BBB integrity following ischemic stroke is regulated by a dynamic balance between protective and disruptive mechanisms orchestrated by ZFPs. KLF family members function synergistically to fortify the paracellular route through tight junction reinforcement. This comprehensive perspective underscores that therapeutic strategies for barrier preservation must simultaneously reinforce protective pathways and inhibit disruptive mechanisms.

Research on ZFPs in hemorrhagic stroke, specifically intracerebral hemorrhage (ICH), has remained relatively limited compared to the ischemic subtype. Nevertheless, emerging evidence indicates their potential involvement in key injury pathways. Studies have reported altered expression of specific ZFPs, such as Gli-similar 2 (Glis2) and ZEB2, in the perihematomal region following ICH. Glis2 was found to be upregulated in neurons and may promote neuronal death through interaction with p75NTR and activation of mitochondrial-dependent apoptotic pathways [268, 269]. Conversely, ZEB2 appears to exacerbate vascular endothelial dysfunction and BBB disruption, potentially via a positive feedback loop involving murine double minute2 (MDM2) [270]. Furthermore, miR-141-3p has been shown to partially attenuate BBB damage by targeting and inhibiting ZEB2 [271]. These preliminary findings suggest that ZFPs may participate in regulating critical injury responses after ICH.

In summary, ZFPs establish a critical molecular network that governs the onset and progression of stroke. In the predominant ischemic stroke subtype, ZFPs drive major upstream risk factors such as hypertension, hyperglycemia, and atherosclerosis. They are also deeply involved in core post-ischemic pathological processes, including oxidative stress, neuroinflammation, programmed cell death, vascular repair responses, and BBB disruption. The functional complexity of stroke pathology is largely attributable to the cell-type specificity and context dependency of ZFPs, as exemplified by the opposing roles of KLF4 in different cell types or ZEB1 across experimental models. While research on hemorrhagic stroke remains at an early stage, ZFPs have been shown to regulate shared pathological mechanisms such as BBB integrity and neuronal fate. Consequently, future therapeutic strategies for stroke should move beyond global activation or inhibition of ZFPs and instead pursue precise, spatiotemporally resolved modulation of specific ZFPs within affected cell populations. This approach could offer a novel direction for simultaneously targeting multiple injury pathways.

#### Alzheimer's disease

The regulatory functions of ZFPs extend from acute cerebral injuries such as stroke to pivotal roles in the gradual pathogenesis of chronic neurodegenerative diseases like Alzheimer's disease (AD). The pathogenesis of AD is defined by the core pathological hallmarks of β-amyloid (Aβ) plaque deposition and the accumulation of neurofibrillary tangles composed of hyperphosphorylated tau protein. These processes initiate a cascade of downstream events that ultimately result in neuronal death and cognitive decline [272]. ZFPs, as critical transcriptional regulators, are deeply implicated throughout the entire continuum of AD. Their involvement originates in upstream genetic regulation and endogenous protective mechanisms. For example, genome-wide association studies identified that variations in the zinc finger homeobox 3 (ZFHX3) gene were associated with cerebrospinal fluid Aβ levels, suggesting its potential role as a genetic determinant influencing Aβ metabolism [273]. Similarly, the transcription factor REST, which activates during early pathological stages, establishes a crucial defensive checkpoint by repressing the expression of multiple pro-degenerative genes, including those encoding γ-secretase and tau kinases. Studies in animal models confirmed that loss of REST accelerated pathology, whereas its overexpression conferred neuroprotection [111]. Notably, however, dysregulated interactions with miR-153-3p, resulting in abnormally elevated REST expression, paradoxically promote the accumulation of toxic proteins, such as amyloid precursor protein (APP), thereby exacerbating neuronal damage [10].

As the disease progresses, ZFPs further contribute to the direct regulation and amplification of Aβ-mediated toxicity. Research demonstrated that p52-ZER6/ZFP398 linked Aβ pathology to oxidative stress by repressing transcription of the antioxidant protein MT3, thereby establishing a pathogenic “transcriptional repression-oxidative stress” axis [95]. Concurrently, downregulation of Yin Yang 1(YY1) has been shown to reduce the expression of its target gene triggering receptor expressed on myeloid cells (TREM2), thereby impairing microglial clearance capacity and anti-inflammatory functions. This impairment exacerbates Aβ deposition and neuroinflammation, creating a self-reinforcing vicious cycle [113]. Furthermore, certain ZFPs themselves serve as biomarkers that reflect disease progression. For instance, significant upregulation of ZFP587B in plasma not only holds diagnostic potential but also links cellular senescence to late-stage neurodegeneration via the senescence-associated secretory phenotype (SASP) [274]. Collectively, these findings elucidate how ZFPs provide molecular links between distinct pathological events in AD, underscoring the complexity of the underlying mechanisms while simultaneously revealing novel potential targets for therapeutic intervention.

#### Parkinson's disease

The core pathology of PD, similar to that of AD, involves the aggregation of misfolded proteins and disrupted proteostasis, primarily targeting α-synuclein and dopaminergic neurons in the substantia nigra [275]. Within this multifaceted pathological framework, ZFPs emerge as critical regulators, influencing disease initiation, progression, and phenotypic specificity.

During disease initiation, ZFPs directly modulate the production and clearance of α-synuclein. For example, ZFP219 has been shown to bidirectionally regulate the expression of the *SNCA* gene, directly affecting α-synuclein synthesis and aggregation [11]. Under physiological conditions, ZFP27 enhances autophagic flux by binding to the promoter of the autophagy gene LC3, thereby promoting α-synuclein clearance and maintaining neuronal homeostasis. However, studies in PD models demonstrated that iron deposition inhibited the expression of insulin-like growth factor 2 (IGF2), leading to the downregulation of ZFP27. This suppression consequently impaired autophagic function and accelerated disease progression [11].

As the pathology advances, ZFPs further exacerbate downstream damage mechanisms, including mitochondrial dysfunction and oxidative stress. ZFP746 (PARIS) was significantly elevated in the plasma of PD patients and correlated inversely with Parkin levels [103]. Functionally, ZFP746 transcriptionally represses peroxisome proliferator-activated receptor gamma coactivator 1-alpha (PGC-1α) and Nrf2-driven antioxidant genes. This repression severely impairs mitochondrial biogenesis and amplifies oxidative stress and neuronal apoptosis [103, 104]. Additionally, research has indicated that ZFP746 is phosphorylated by the non-receptor kinase c-Abl, a modification that mediates α-synuclein-induced degeneration of dopaminergic neurons [103].

Regarding endogenous neuroprotection, certain ZFPs perform crucial physiological roles, and their functional loss contributes to pathogenesis. For instance, ZFP184 inhibits the transcription of interleukin enhancer binding factor 3 (ILF3) by binding to its promoter, which relieves ILF3’s inhibitory effect on miR-7 biogenesis. The subsequent upregulation of miR-7 reduces α-synuclein aggregation, protecting dopaminergic neurons and alleviating motor deficits [276]. Similarly, ZFP91 specifically binds to the G-quadruplex (G4) DNA sequence within the SVA retrotransposon associated with X-linked dystonia-Parkinsonism (XDP), inhibiting its transcriptional activity and preventing dysregulation of *TAF1* gene expression. The age-related decline in ZFP91 expression reduces this inhibitory effect, thereby driving the progressive pathology of XDP [277].

Conversely, abnormal expression of other ZFPs can amplify neural injury. Knockdown of ZNRF2, for example, activates the mTOR signaling pathway, exacerbating neuroinflammation, dopaminergic neuron degeneration, and motor dysfunction [278]. Furthermore, loss-of-function mutations in the *PARK2* gene, which encodes the E3 ubiquitin ligase Parkin, lead to the accumulation of ZFP746 in patient brains. Genetic knockout of ZFP746 was found to alleviate neurodegeneration in models, confirming its pathogenic function [11].

In summary, ZFPs form a complex and precise regulatory network in PD. They influence the metabolic balance of the core pathological protein α-synuclein at an upstream level and profoundly participate in amplifying downstream neural damage by disrupting key pathways involving mitochondrial function, antioxidant defense, and neuroinflammation. The abnormal accumulation of specific members (e.g., ZFP746) directly drives neural injury, while the functional loss of others (e.g., ZFP184, ZFP91) compromises essential neuroprotective mechanisms. This dual role of pathogenic potentiation and protective attenuation establishes the ZFP network as a critical intrinsic hub that integrates and propels multiple pathological processes in PD**.**

#### Amyotrophic lateral sclerosis

Distinct from the specific protein-centric pathologies of AD and PD, ALS is characterized by core pathological processes involving disrupted protein homeostasis, widespread dysregulation of RNA metabolism, and persistent neuroinflammation. These interconnected mechanisms collectively drive the degeneration of motor neurons [279]. Within this complex pathogenic network, ZFPs serve as pivotal regulatory factors, fulfilling multifaceted roles spanning aberrant RNA processing to the failure of neuroprotective mechanisms.

In the realm of RNA metabolism and toxicity, ZFPs directly engage in processing abnormal nucleic acid structures and modulating toxic products. ZFP106 reduces the synthesis of toxic dipeptide repeat proteins by inhibiting the repeat-associated non-AUG (RAN) translation of the C9orf72 GGGGCC repeat expansion. It also specifically binds to and remodels RNA containing G-quadruplex structures, such as G4C2 repeat sequences, thereby neutralizing their pathogenicity [280]. Conversely, mutations in fused in sarcoma (FUS) disrupt normal RNA binding and metabolism, leading to aberrant nucleocytoplasmic transport and the formation of cytotoxic aggregates that drive neuronal injury [281].

At the level of transcriptional regulation and neuroprotective imbalance, the pathological environment impairs the normal transcriptional functions of ZFPs. YY1, via its zinc finger domain, activates target genes such as Fuzzy to maintain synaptic stability. However, GGGGCC repeat RNAs associated with ALS competitively bind to this domain, blocking its transcriptional activity and consequently leading to the failure of neuroprotective pathways [114]. Furthermore, in sporadic ALS, the downregulation of the long non-coding RNA ZEB1-AS1 disrupts the precise regulatory loop it forms with the hsa-miR-200c/ZEB1/β-catenin axis, ultimately impairing neuronal differentiation and synaptic integrity [282].

In summary, ZFPs constitute an integral regulatory network in ALS. Their functions range from direct management of aberrant RNA metabolism and toxicity, as exemplified by ZFP106 and FUS, to the maintenance of transcriptional programs essential for neuronal homeostasis, as seen in the YY1 and ZEB1-AS1-related pathways. These functions collectively propel ALS progression and underscore the pivotal regulatory role of ZFPs in the disease mechanism.

#### Epilepsy

Extending beyond chronic degenerative diseases, ZFPs act as core modulators in paroxysmal disorders like epilepsy, which is fundamentally driven by an imbalance in neuronal network excitability and pathological synaptic remodeling [283]. Recent studies have demonstrated that ZFPs play central regulatory roles in this complex network through transcriptional control, epigenetic modification, and signaling pathway integration.

Genetic analyses have revealed significant enrichment of epilepsy-associated single nucleotide polymorphisms in ZFP gene regions, establishing this family as a genetic susceptibility factor for epilepsy [284]. Expression profiling further identified the downregulation of several KRAB-ZFP genes following seizures, potentially contributing to pathogenesis through derepression of excitability-related genes [285]. KLF family members exhibit distinct functional roles. For example, KLF4 was found to promote synaptic plasticity-related protein expression via the STAT3 pathway and to reduce seizure phenotypes in mouse models, thereby indicating neuroprotective potential [33]. Conversely, KLF6 was upregulated in the hippocampus following status epilepticus and colocalized with heat shock protein 47 (HSP47) in astrocytes, suggesting its involvement in post-epileptic tissue repair and scar formation [38]. Interventional studies have confirmed that REST inhibition reduces seizure frequency and severity [286]. Clinical investigations further established serum REST levels as a potential biomarker, showing significant elevation in epilepsy patients with cognitive impairment [112]. Additionally, intervention experiments demonstrated that Smo inhibitors reduced epileptiform neuronal activity by inhibiting Gli1 transcriptional activity [123].

In summary, ZFPs play crucial roles in core epileptic processes, such as modulating neuronal excitability, glial cell responses, and intracellular signaling, through multi-level molecular mechanisms. Existing evidence indicates that specific ZFP family members, particularly REST, KLF4, KLF6, and Gli1, are involved in the pathogenesis of epilepsy and also influence disease progression and treatment response, thereby holding promise as biomarkers and therapeutic targets. Interventions targeting ZFPs may offer a strategy to multi-dimensionally regulate epileptic networks and provide novel avenues for ameliorating associated neurological deficits.

#### Depression

The spectrum of CNS disorders extends beyond motor and cognitive impairments to include affective and behavioral dysfunctions. Depression, a prevalent mental disorder and a leading global cause of disability, is characterized primarily by persistent low mood, diminished interest, and anhedonia, often accompanied by a constellation of somatic symptoms [287]. The pathogenesis of depression is multifaceted, involving dysregulation of neurotransmitter systems, neuroinflammation, aberrant stress responses, and epigenetic modifications. Within this complex pathophysiological framework, ZFPs emerge as critical molecular orchestrators, functioning through distinct regulatory pathways.

In the regulation of monoaminergic neurotransmission, direct binding of Zkscan4/ZFP427 to the 5-hydroxytryptamine receptor 2a (Htr2a) promoter, coupled with recruitment of the glucocorticoid receptor, leads to the formation of a repressive complex. This complex suppresses Htr2a transcription, thereby maintaining excitatory synaptic transmission in the hippocampus [96]. Depressive phenotypes are further modulated by ZFP326 through a dual mechanism: its genetic polymorphism influences antidepressant sensitivity, while its downregulation enhances serotonin expression and attenuates dopamine depletion [288]. Beyond neurotransmitter systems, specific ZFPs are implicated in neuroinflammatory components of depression. Notably, a pro-inflammatory phenotype in glial cells is promoted by GATA1 via transcriptional activation of immune-related genes [143]. Concurrently, MORC1 mRNA is detected in key mood-regulatory brain regions, including the prefrontal cortex, nucleus accumbens, and hippocampus, suggesting its potential role as a mediator of early-life stress effects on depression [289]. Regarding epigenetic regulation and genomic stability, upregulation of KRAB zinc finger proteins (KZFPs) has been observed in the brains of individuals with major depressive disorder. This upregulation coincides with a broad suppression of transposable element transcription, suggesting that pathological depression may involve excessive repression of transposable element activity by KZFPs [290].

Collectively, these findings establish ZFPs as multifunctional regulators of depression pathophysiology, operating across neurotransmitter systems, neuroinflammatory cascades, and stress response pathways. The mechanistic diversity of ZFP involvement, ranging from direct transcriptional repression to epigenetic modulation and signaling pathway integration, underscores their potential as both diagnostic biomarkers and therapeutic targets. Future investigations focusing on ZFP-specific interventions may yield novel treatment strategies that address the multifaceted nature of depression.

**Fig. 4:**
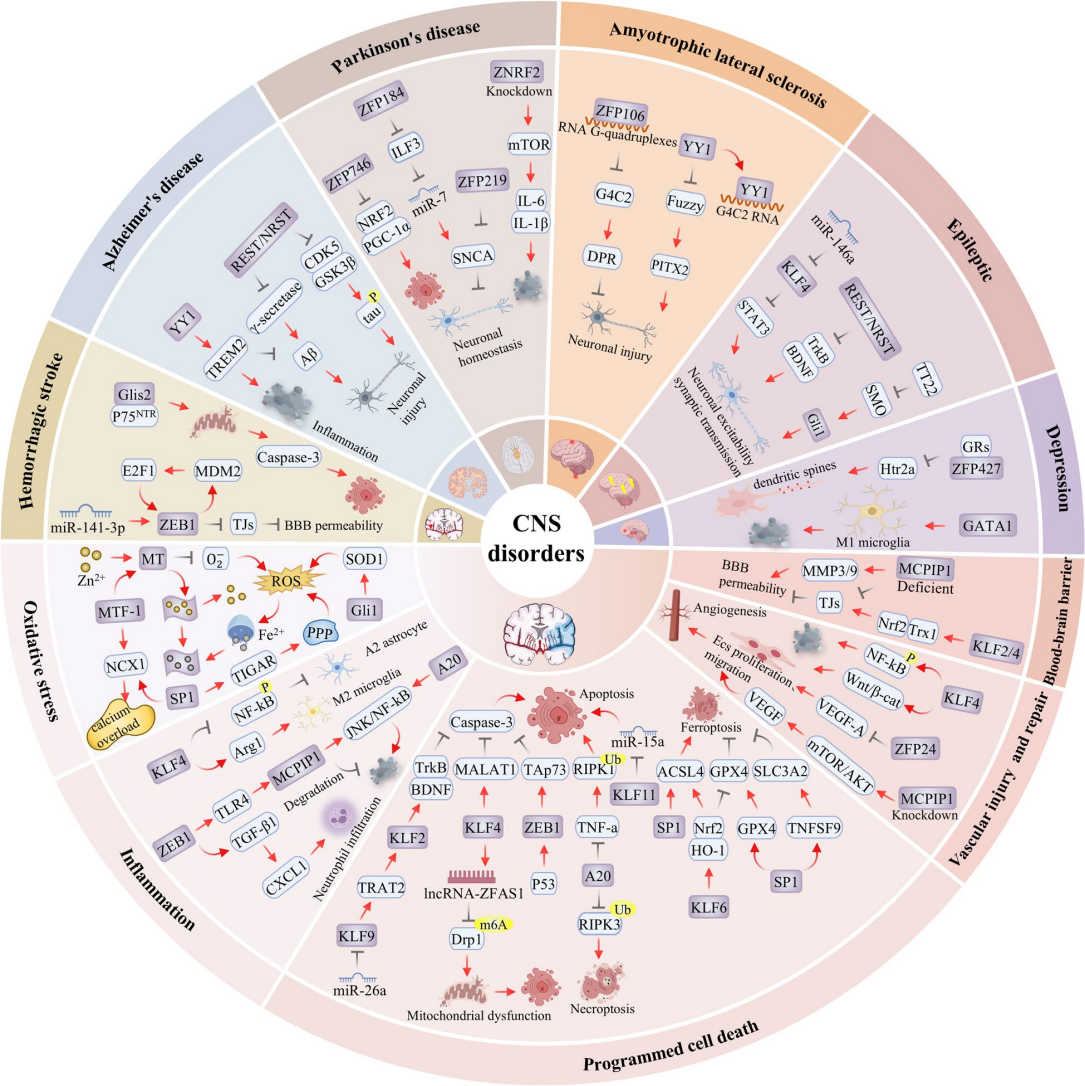
Role of ZFPs in CNS disorders. ZFPs act as central molecular regulators across major central nervous system disorders, with their multifaceted role in ischemic stroke receiving focused elaboration. In this pathological context, ZFPs function as master regulators of programmed cell death, transcriptionally coordinating ferroptosis, apoptosis, and necroptosis to determine cell fate. They simultaneously orchestrate integrated neuroprotective and vascular repair programs by modulating oxidative stress, inflammatory responses, angiogenic signaling, and BBB integrity. Beyond ischemic stroke, the representation highlights the disease-specific involvement of ZFPs in the characteristic pathological mechanisms, including protein aggregation, inflammation, and neuronal loss, underlying hemorrhagic stroke, Alzheimer's disease, Parkinson's disease, amyotrophic lateral sclerosis, epilepsy, and depressive disorders, collectively underscoring their pivotal position in mediating diverse neuropathological cascades

Based on the findings summarized in this section, the role of the ZFP family in CNS disorders is illustrated in Fig. 4. ZFP family mediate their multifaceted roles in CNS disorders largely through their function as master transcriptional regulators, orchestrating comprehensive gene programs related to cell fate determination, inflammatory signaling, redox homeostasis, and neurovascular remodeling.

### Genetic disorders

**Fig. 5:**
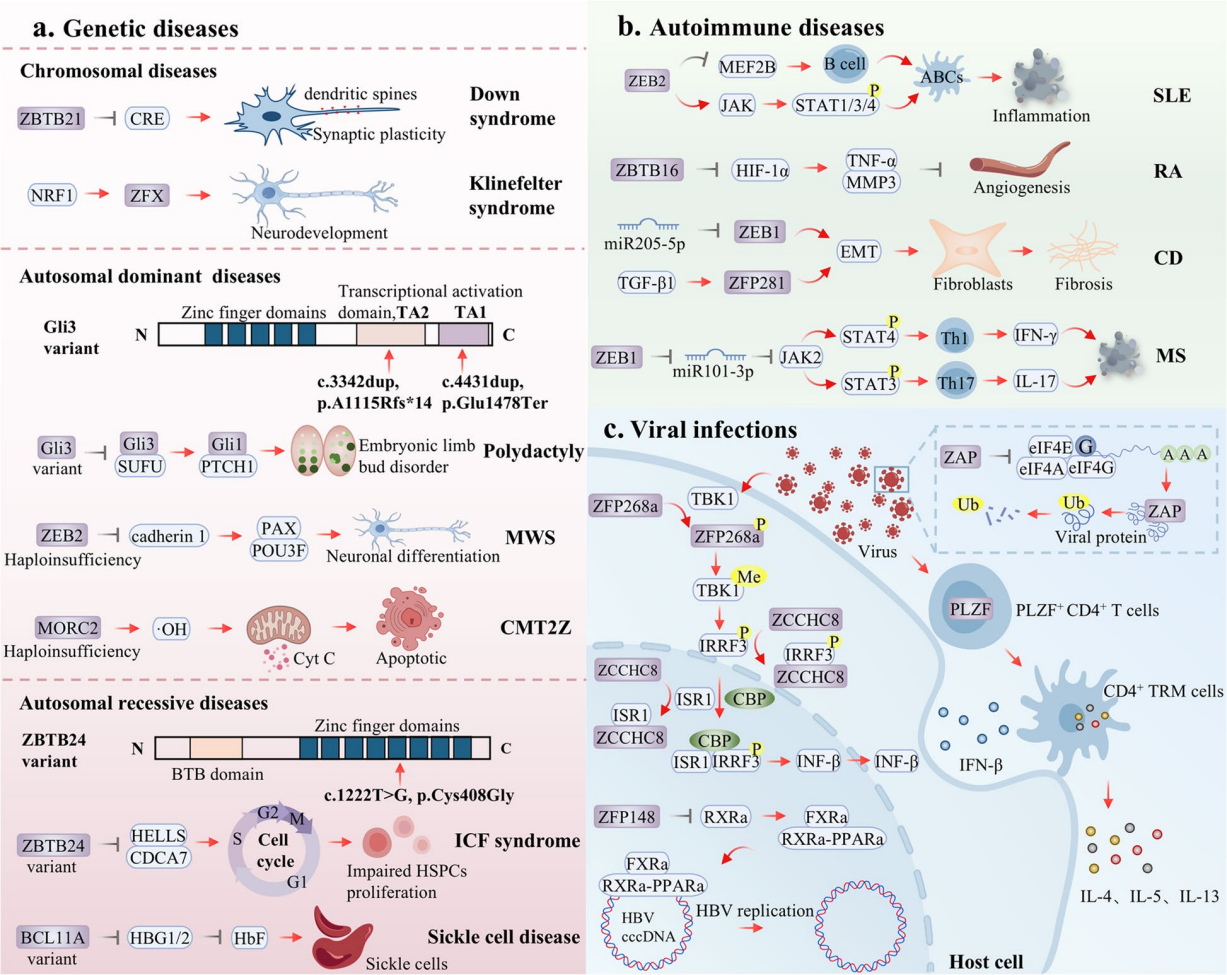
Role of ZFPs in genetic disorders, autoimmune diseases, viral infections. Abbreviations: (MWS: Mowat-Wilson syndrome; CMT2Z: Charcot-Marie-Tooth disease type 2Z; ICF syndrome: immunodeficiency, centromeric instability, and facial anomalies syndrome; SLE: systemic lupus erythematosus; RA: rheumatoid arthritis; CD: Crohn's disease; MS: multiple sclerosis; ABCs: age-associated B cells; HSPCs: hematopoietic stem and progenitor cells)

Dysregulation of ZFPs function contributes not only to acquired pathological states such as cancer and neurological disorders but also, through genetic variations in their encoding genes, underlies numerous congenital diseases. As critical interpreters and executors of DNA sequences, even subtle alterations in ZFP activity can directly translate into significant developmental and physiological abnormalities. Genetic diseases are broadly classified into categories such as chromosomal disorders, monogenic diseases, and genomic imprinting disorders, with ZFPs mediating a wide range of associated pathological phenotypes through multi-layered mechanisms (Fig. 5).

In chromosomal disorders, specific cognitive deficits observed in Down syndrome (trisomy 21) have been associated with an increased dosage of the *ZBTB21* gene, which inhibits cAMP response element-dependent gene expression [291]. Furthermore, in Klinefelter syndrome, expression of the X-linked *ZFX* gene is regulated by the autosomal transcription factor NRF1, thereby revealing a complex gene-regulatory network operating under aneuploid conditions [292].

Among monogenic hereditary diseases, autosomal dominant forms primarily involve gain-of-function or haploinsufficiency mechanisms. Truncating mutations in *GLI3* were shown to directly cause polydactyly [293], specially, truncating *GLI3* mutations abolished SUFU binding and compromised the transcriptional activation of Shh target genes (e.g., *PTCH1*, *Gli1*). This led to a disruption in limb bud patterning and caused polydactyly [293, 294]. Additionally, ZEB2 haploinsufficiency was identified as the underlying cause of multisystem developmental malformations in Mowat-Wilson syndrome. During neural development, this defect was shown to reduce the repressor activity of ZEB2 at the promoters of target genes such as E‑cadherin. This disruption impaired the neurodevelopmental regulatory network coordinated by key transcription factors including PAX and POU3F, ultimately leading to temporally and lineage-specific dysregulation of neuronal differentiation [295]. Similarly, haploinsufficiency of *MORC2* drove the progression of Charcot-Marie-Tooth disease type 2Z (CMT2Z) by inducing oxidative stress and mitochondrial dysfunction [135].

Autosomal recessive diseases are predominantly caused by loss-of-function mutations in ZFPs or by their complete absence. Loss-of-function mutations in BCL11 transcription factor A (*BCL11A*) prevented the developmental switch from fetal hemoglobin to adult hemoglobin, and sustained expression of this transcription factor has demonstrated therapeutic potential for hemoglobinopathies such as sickle cell disease [296, 297]. Studies confirmed that mutations in *ZBTB24* impaired hematopoietic stem and progenitor cells (HSPCs) proliferation, primarily through disruption of the cell division cycle-associated protein 7 (CDCA7)/Helicase lymphoid-specific (HELLS) chromatin remodeling axis during S-phase, leading to immunodeficiency, centromeric instability, and facial anomalies (ICF) syndrome [61]. Biallelic variants in *ZFP526* and *ZFP335* lead to neurodevelopmental disorders, including microcephaly [90, 298].

In X-linked genetic disorders, pathogenic variants in *ZMYM3* and *ZMYND8* have been consistently reported to cause neurodevelopmental disorders through chromatin modification disruption, with phenotype severity closely associated with X-chromosome inactivation mechanisms [141, 299]. Genomic imprinting disorder research has revealed that *ZFP274* participates in Prader-Willi syndrome pathogenesis by mediating *SNORD116* gene-cluster epigenetic silencing [81].

In summary, ZFPs serve as molecular converters that transform diverse genetic alterations into specific pathological outcomes across hereditary diseases. They interpret abnormal gene dosage in chromosomal disorders, execute haploinsufficiency or gain-of-function effects in monogenic diseases, and mediate allele-specific regulation in imprinting disorders. Ultimately, ZFPs provide a framework for understanding how genetic information is processed into phenotypic outcomes, highlighting potential intervention points for modifying disease expression.

### Autoimmune diseases

Transitioning from endogenous genetic defects to diseases driven by the immune system's erroneous attack on self-tissues, ZFPs constitute indispensable regulatory hubs within autoimmune pathological networks. Autoimmunity is initiated in genetically susceptible individuals when environmental triggers lead to a breakdown of immune tolerance, culminating in immune cell infiltration of target organs and chronic tissue damage. Throughout this pathological continuum, ZFPs exert central transcriptional and post-transcriptional control, functioning in maintaining immune cell quiescence, establishing activation thresholds, and regulating terminal inflammatory responses (Fig. 5).

Substantial evidence has shown that genetic polymorphisms among ZFPs constitute an important component of autoimmune disease susceptibility. For instance, in systemic lupus erythematosus (SLE), specific single-nucleotide polymorphisms (SNPs) in *IKZF1* and *ZFP76* (such as rs4132601 and rs10947540) were found to alter the expression or function of immune-related molecules including CD40 ligand, thereby increasing disease risk [63, 300]. Similarly, *ZBTB* was shown to bind risk variants in the *IRF5* enhancer region (rs4728142), contributing to SLE pathogenesis [301]. In multiple sclerosis (MS), variants in *IKZF3* (rs12946510) disrupted immune homeostasis through eQTL-mediated dysregulation of *ORMDL3*/*GSDMB* expression [65]. Collectively, these genetic variations establish the initial intrinsic conditions for immune system imbalance.

Following the breakdown of immune tolerance, the homeostatic functions of ZFPs become dysregulated. In rheumatoid arthritis (RA), downregulation of the protective factor ZBTB16 relieved inhibition of the HIF-1α pathway, promoting pathological angiogenesis, while reduced expression of IKZF2 and IKZF3 compromised regulatory T cell stability, collectively contributing to disease onset [56, 64].

As inflammatory signals amplify, activated immune cells infiltrate target organs, where ZFPs directly drive tissue-specific damage. In SLE, abnormal overexpression of ZEB2 in senescent CD4 + T cells drove cytotoxicity and excessive B cell assistance, directly exacerbating tissue damage [12]; ZEB2 also promoted age-associated B cells (ABCs) differentiation via the JAK-STAT pathway, further facilitating lupus pathogenesis [302]. Concurrently, upregulation of ZEB1 and ZFP281 accelerated intestinal fibrosis by promoting collagen deposition in Crohn’s disease (CD) [86, 303]. Within the central nervous system of MS patients, abnormal activation of ZEB1 promoted pathogenic Th1/Th17 responses through JAK2/STAT3/STAT4 signaling [47].

In summary, ZFPs critically regulate the pathological continuum of autoimmune diseases, spanning from genetic susceptibility to terminal tissue damage. Their polymorphic variants set the initial threshold for immune dysregulation by altering the expression of key immunomodulatory molecules. Upon tolerance breakdown, the homeostatic functions of protective ZFPs are impaired, accelerating disease initiation. As inflammation escalates, ZFPs are co-opted to drive pathogenic immune cell differentiation and directly execute programs of tissue infiltration and fibrosis in target organs. Thus, ZFPs function as dynamic regulators that not only safeguard immune equilibrium but also, when dysregulated, actively propel disease progression across multiple stages, highlighting their potential as stage-specific therapeutic targets.

### Viral infections

The immune system functions both as a source of self-directed pathology and as the primary defense against external threats. In the context of viral infection, ZFPs serve as critical cellular regulators, establishing a coordinated defensive network by directly targeting viral components, modulating immune responses, and altering host cell permissiveness (Fig. 5).

During the viral replication phase, ZFPs can directly intervene in the viral life cycle. For instance, ZAP recognizes CpG-rich sequences in viral RNA via its N-terminal zinc finger domain, recruits cellular cofactors, and inhibits viral mRNA translation, thereby effectively blocking viral gene expression and replication [131]. Additionally, ZFP148 has been shown to restrict viral replication by downregulating the transcription factor *RXRα*, which consequently suppresses the transcriptional activity of hepatitis B virus covalently closed circular DNA (cccDNA) [77].

In the context of immune regulation, ZFPs modulate the interferon pathway in a bidirectional manner. For example, Tank-binding kinase 1 (TBK1)-mediated phosphorylation of ZFP268a and subsequent methylation of TBK1 by SET domain containing 4 (SETD4) constitute a positive regulatory mechanism for interferon signaling [13]. Conversely, ZCCHC8 was found to inhibit type I interferon production by interfering with interferon regulatory factor 3 (IRF3) binding to its co-activator CREB-binding protein (CBP) and to the interferon-stimulated response element (ISRE) [137]. This bidirectional regulatory mechanism underscores the pivotal role of ZFPs in maintaining immune homeostasis.

Furthermore, ZFPs influence viral infection processes by altering host cell gene expression. *GATA6* was found to directly bind to the *ACE2* gene promoter and upregulate its expression, thereby enhancing cellular susceptibility to severe acute respiratory syndrome coronavirus 2 (SARS-CoV-2) infection [145]. In a respiratory syncytial virus infection model, the promyelocytic leukemia zinc finger (PLZF) was observed to drive the differentiation of CD4 + tissue-resident memory T cells, modulating Th2-type immune responses and pulmonary inflammation [57].

Collectively, ZFPs establish a synergistic antiviral defense network through direct viral targeting, immunoregulatory functions, and host cell state modulation. Further elucidation of these mechanisms will provide novel molecular targets and theoretical foundations for developing broad-spectrum antiviral therapeutics.

## Therapeutic strategies for targeting ZFPs

ZFPs have emerged as highly promising therapeutic targets due to their pivotal roles in human diseases, yet their targeted development is confronted with inherent challenges. These proteins typically lack deep binding pockets recognizable by conventional small molecules, and the high structural homology among family members renders selective intervention difficult. Furthermore, the functional complexity and context dependency of ZFPs further exacerbate the difficulty of precise modulation. Currently, research strategies are advancing along multiple dimensions, including the development of novel small molecules capable of indirectly modulating ZFP function or inducing its degradation, the utilization of protein degradation technologies to directly eliminate target ZFPs, and the root-correction of their dysfunction via gene editing and regulation technologies. Collectively, these strategies form a diversified intervention system for ZFP targeting.

### Challenges in targeting transcription factors

Given the central role of ZFPs in disease pathogenesis, targeting their function has emerged as a compelling therapeutic paradigm. However, this approach faces considerable challenges rooted in protein structure. Traditional small-molecule drugs typically function by binding with high affinity to well-defined “pockets” or “clefts” on target protein surfaces. These sites often feature hydrophobic character and distinct geometric boundaries. In contrast, the core functional interfaces of most transcription factors, including ZFPs, tend to be expansive, flat, and predominantly hydrophilic. For example, ZFPs engage DNA through their DNA-binding domains or interact with transcriptional cofactors via protein–protein interaction surfaces. These interfaces generally lack the deep, structurally confined cavities required for potent and selective small-molecule engagement [304]. This structural characteristic forms the basis for the classical “undruggability” of ZFPs. Consequently, despite their central roles in disease pathogenesis, drug discovery efforts targeting ZFPs have historically faced significant obstacles.

Beyond the general druggability challenges common to transcription factors, ZFPs present additional unique complexities. First, the high degree of sequence homology within ZFP families complicates selectivity achievement. For instance, the zinc finger domains within the KLF family share considerable similarity [14], making it exceptionally difficult to design small molecules that modulate a single member without affecting homologous proteins and thereby raising the risk of off-target effects and toxicity. Second, target validation proves particularly demanding. Even when candidate compounds are identified through screening, rigorous multidisciplinary validation is essential. It must be established that their biological effects originate from direct and specific binding to the intended ZFP, followed by a demonstration of the expected protective or therapeutic activity in relevant in vitro and in vivo models [305]. This process typically requires integrating biophysical, structural, and functional assays, which presents high technical and temporal barriers.

Perhaps most critically, ZFPs exhibit profound functional complexity and context-dependence. They frequently occupy nodal positions within transcriptional networks, where a single ZFP can exert opposing effects depending on the downstream pathway, cell type, or disease stage. In ischemic stroke, for example, the transcription factor *SP1* promotes antioxidant defense by activating *TIGAR*, thereby conferring protection [119]. Simultaneously, SP1 drives ferroptosis via the ACSL4 pathway, exacerbating injury [252]. This illustrates SP1’s capacity to regulate counteracting processes within the same pathology. ZEB1 demonstrates a cell-type-specific dual role: in neurons, it exhibits both anti-apoptotic activity and suppressibility linked to neuroprotection [255, 256]; in microglia, it primarily functions as an anti-inflammatory regulator [50]. In acute myeloid leukemia (AML), ZEB1 also displays functional multiplicity. It can suppress the malignant potential of leukemia stem cells by modulating hematopoietic self-renewal, apoptosis, and differentiation [306], yet it can also drive tumor invasiveness and promote immune evasion to accelerate disease progression [307]. Furthermore, ZEB1 has been reported to exert oncogenic functions across various solid tumors, including those of the lung, breast, colon, and liver [43–46]. This high degree of context-dependent plasticity necessitates precise consideration of the cellular and pathological setting when designing ZFP-targeted interventions, which substantially increases the difficulty of therapeutic strategy development.

In summary, ZFPs present fundamental challenges at the structural, selectivity, and functional complexity levels, which have long classified them as intractable targets. However, the emergence of novel therapeutic modalities is driving a paradigm shift in this field. The subsequent sections summarizes recent representative cases in which specific ZFPs have been successfully targeted using innovative strategies, including small-molecule inhibition, protein degradation, and gene editing. These advances not only confirm the feasibility of targeting ZFPs but also provide a practical framework for systematically addressing the challenges outlined above.

### Small molecule inhibitors and activators

Although directly targeting transcription factors, especially ZFPs, presents formidable challenges due to their lack of classical drug-binding pockets and flat interaction interfaces, significant progress has been made. Advances in high-throughput screening, structural biology, and rational drug design have enabled the development of multiple strategies for precise modulation. As summarized in Table 2, these include allosteric inhibitors, protein degraders, upstream pathway modulators, and functional agonists, which have demonstrated therapeutic potential across various disease models. These achievements not only validate the feasibility of targeting ZFPs but also provide critical proof-of-concept and direction for future development.

**Table 2:** Small molecule inhibitors and activators targeting ZFPs

Disease	Interventions	Targets	Regulatory factors	Effects or functions	NCT numbers	Ref
HCC	Dihydroartemisinin (DHA)	MAZ↓	TRIM50↑, p62↑, Beclin 1↑	Induction of autophagy	Not Applicable	[308]
	GANT61	Gli1↓	FoxM1↓, CyclinD1↓, Bcl-2↓	Inhibition of cellular proliferation and induction of cell cycle arrest	Not Applicable	[309]
	Bufalin	E2F2-ZFP91↑	E2F2↓, c-Myc↓, CCNE1↓, CCNE2↓, MCM5↓, CDK1↓	Inhibition of tumor growth, induction of E2F2 degradation, and down-regulation of oncogene transcription	Not Applicable	[310]
TNBC	Curcumin	Gli1↓	PTCH1↓, Gli2↓, SMO↓, E-cadherin↑, vimentin↓	Inhibition of EMT and stem cell properties	Not Applicable	[311]
	β, β-Dimethylacryloyl Alkannin	Gli1↓	AKT(Ser473) ↓	Inhibition of cellular proliferation and induction of apoptosis	Not Applicable	[312]
Breast Cancer	Rolipram	Gli1↓	PKA↑, p-GSK3β↓	Inhibition of the Hedgehog signaling pathway	Not Applicable	[313]
CRC	Resveratrol (RSV)	ZEB1↓	m^6^A RNA modification↓, E-cadherin↑, N-cadherin↓, vimentin↓	Inhibition of EMT	Not Applicable	[314]
	Curcumin	ZBTB7A↓	CD95↓, JNK2↓	Induction of cell death	Not Applicable	[315]
	Daunorubicin	Gli1↓	p53↑, β-TrCP↑	Promotion of apoptosis	Not Applicable	[316]
NSCLC	Metformin	Snail↓	GDF15↑, ATF4↑, CHOP↑, AMPK↑	Inhibition of epithelial-mesenchymal transition	Not Applicable	[317]
	GANT-61	Gli1↓	bFGF↓, FGFR1↓, FGFR2↓	Inhibition of tumor angiogenesis	Not Applicable	[318]
HSV-1	Mithramycin A	Sp1↓, Sp3↓	ICP4↓, VP16↓	Inhibition of viral transcription	Not Applicable	[319]
PD	Chiisanoside	ZFP746↓	miR-181a↓, Parkin↑, PGC-1α↑, NRF1↑, TFAM↑	Promotion of mitochondrial biogenesis and anti-apoptotic effects	Not Applicable	[320]
AD	Tolfenamic Acid	SP1↓	miR-15a-5p↓, NOS3↑	Promotion of mitochondrial biogenesis and anti-apoptotic effects	Not Applicable	[321]
Cerebral Ischemia	Polydatin	Gli1↑	NF-κB↓, SOD1↑, Ptch1↑	Anti-inflammatory effects, antioxidant stress protection, and preservation of the blood–brain barrier	Not Applicable	[322]
Cerebral Edema	RSV	SP1↓	SUR1↓, AQP4↓	Alleviation of cerebral edema and preservation of the blood–brain barrier	Not Applicable	[323]
IS	Ramelteon	KLF2↑	E-selectin↓	Inhibition of monocyte-endothelial cell adhesion	Not Applicable	[324]
	Melittin	MCPIP1↑	IL-1β↓, IL-6↓, TNF-α↓	Anti-inflammatory effects	Not Applicable	[325]
	Minocycline	MCPIP1↑	TNFα↓, IL-1β↓, IL-6↓, MCP-1↓	Anti-inflammatory effects	Not Applicable	[326]
	Huoluo Xiaoling Pellet(HXP)	MCPIP1↑	TNF-α↓, IL-1β↓, IL-6↓, iNOS↓	Promotion of microglial M2 polarization and anti-inflammatory effects	Not Applicable	[327]
	Tetramethylpyrazine	MCPIP1↑	TNF-α↓, IL-1β↓, IL-6↓, MCP-1↓, MMP-9↓	Anti-inflammatory effects with preservation of the blood–brain barrier	Not Applicable	[328]
	Clematichinenoside	A20/TNFAIP3↑	iNOS↓, ICAM-1↓, NF-κB↓, TNF-α↓, IL-1β↓	Anti-inflammatory effects with preservation of the blood–brain barrier	Not Applicable	[329]
SCD	Busulfan, Plerixafor	BCL11A/ZFP856	HbF↑	Enhancement of total hemoglobin synthesis	NCT03653247	[330]
AML	Vismodegib	Gli1↓	UGT1A↓	Inhibition of glucuronidation process	NCT02073838	[331]
RA	Methotrexate	ZPR1/ZFP259	Not Applicable	Biomarkers predictive of treatment efficacy	NCT01034137	[332]
Hyperlipidemia	Simvastatin	ZNF542P↓	Not Applicable	Elevation of intracellular cholesterol ester accumulation	NCT00451828	[333]

*IS* ischemic stroke, *HCC* hepatocellular carcinoma, *DHA* Dihydroartemisinin, *TRIM50* tripartite motif containing 50, *EMT* epithelial-mesenchymal transition, *TNBC* triple-negative breast cancer, *GSK3β* glycogen synthase kinase 3β, *CRC* colorectal cancer, *E-cadherin* epithelial cadherin, *N-cadherin* neuro-cadherin, *RSV* Resveratrol, *NSCLC* non-small cell lung cancer, *GDF15* growth differentiation factor 15, *ATF4* activated transcription factor 4, *CHOP* C/EBP-homologous protein, *AMPK* AMP-activated protein kinase, *HSV-1* human alphaherpesvirus 1, *PD* Parkinson's disease, *PGC-1α* proliferator-activated receptor gamma coactivator-1-alpha, *NRF1* nuclear respiratory factor 1, *TFAM* mitochondrial transcription factor A, *AD* Alzheimer's disease, *NOS3* nitric oxide synthase 3, *SUR1* Sulfonylurea receptor 1, *AQP4* aquaporin 4, *HXP* Huoluo Xiaoling Pellet, *SCD* Sickle Cell Disease, *AML* Acute Myeloid Leukemia, *RA* Rheumatoid arthritis, *HUSH* Human silencing hub

These strategies have been successfully applied in diverse therapeutic contexts. For instance, direct transcriptional inhibition remains a classic and effective approach. GANT61 suppressed proliferation and angiogenesis in hepatocellular carcinoma and non-small cell lung cancer by inhibiting Gli1 [309, 318]. Mithramycin A suppressed viral infections through competitive inhibition of the SP1/SP3 DNA-binding-mediated transcriptional activation functions [319]. In breast cancer, curcumin and β, β-dimethylacryloyl alkannin inhibited tumor progression by targeting the Gli1 pathway [311, 312]. Additionally, resveratrol reduced cerebral edema and protected the blood–brain barrier by downregulating SP1 [323].

Beyond direct inhibition, modulating ZFP protein stability offers another effective strategy. Daunorubicin and the natural product bufalin inhibited tumor growth by promoting Gli1 ubiquitination-mediated degradation or by acting as molecular glues to induce E2F2 degradation, respectively [310, 316]. A similar degradation mechanism has been applied in neurological disorders. Chiisanoside, for instance, restored mitochondrial function by degrading ZFP746 [320].

Indirect modulation of ZFP function by intervening in upstream signaling pathways also shows therapeutic potential. Rolipram inhibited breast cancer by promoting Gli protein degradation via the cAMP/PKA axis [313], while metformin suppressed lung cancer metastasis by inhibiting Snail through AMPK signaling [317]. In CRC, curcumin and resveratrol exerted therapeutic effects by inhibiting ZBTB7A and downregulating ZEB1, respectively [314, 315].

Furthermore, upregulating protective ZFPs provides novel insights for treating ischemic and inflammatory diseases. In stroke models, ramelteon protected the vascular endothelium by upregulating KLF2 [324], whereas minocycline, melittin, and Huoluo Xiaoling Pellet exerted anti-inflammatory and neuroprotective effects by upregulating MCPIP1 [325–327]. Polydatin produced neuroprotective effects by activating endogenous Gli1 expression [322], and clematichinenoside alleviated inflammatory injury via the A20/NF-κB pathway [329]. In AD, tofenacic acid derivatives offered a new paradigm for directly regulating transcriptional programs by competitively inhibiting SP1-DNA binding [321].

In summary, the targeting of ZFPs has evolved into a multi-faceted therapeutic framework that includes direct inhibition, targeted degradation, indirect pathway modulation, and functional activation. These strategies have demonstrated considerable promise across diverse disease models, offering practical solutions to the long-standing challenge of drugging transcription factors. However, limitations persist for traditional small-molecule approaches against ZFPs that lack well-defined binding pockets. This challenge has motivated a shift toward a more fundamental interventional paradigm: instead of modulating protein activity, the objective is to achieve its complete removal from the cell. Targeted protein degradation technologies have thus emerged as a direct and strategic response to this unmet need.

### Protein degradation technologies

Targeted Protein Degradation (TPD) constitutes a paradigm shift in drug development, fundamentally extending the scope of traditional pharmacological strategies. In contrast to classical small-molecule inhibitors or activators, TPD is designed to harness the endogenous ubiquitin–proteasome system for the complete elimination of pathogenic proteins. This strategic pivot enables the targeting of proteins that lack defined active sites, including transcription factors and scaffolding proteins, thereby addressing targets previously considered undruggable [334]. The technology demonstrates several distinct advantages: it accesses challenging protein targets through a dual-selection mechanism involving E3 ligase and tissue specificity; it induces sustained pharmacological effects that persist beyond protein resynthesis; and it effectively circumvents resistance mechanisms associated with point mutations by eliminating rather than inhibiting pathogenic proteins [335, 336].

In this evolving paradigm, two principal strategies have emerged: proteolysis-targeting chimeras (PROTACs) and molecular glues. PROTACs function as heterobifunctional molecules that facilitate target protein ubiquitination and degradation by bridging them to E3 ubiquitin ligases [337]. Notably, pomalidomide, a frequently utilized E3 ligase recruiter in PROTAC design, inherently exhibits molecular glue properties that can prompt unintended ZFP degradation. Strategic chemical modification at pomalidomide’s C5 position has been shown to selectively attenuate zinc finger binding while preserving degradation efficacy against intended targets, thereby minimizing off-target effects [337].

Substantial progress in ZFP degradation has been achieved through the rational optimization of established platforms. Park et al. employed structure-guided design to develop DEG-35, a lenalidomide-based degrader capable of simultaneously targeting both casein kinase 1 alpha (CK1α) and IKZF2 at nanomolar concentrations in acute myeloid leukemia models. Their subsequent development of DEG-77, a solubility-enhanced analog, demonstrated improved in vivo efficacy and selectivity [338]. Further advancing this field, NVP-DKY709 emerged as a clinical-stage molecular glue that selectively recruits CRBN to degrade IKZF2 while sparing IKZF1/3, thereby attenuating Treg-mediated immunosuppression and overcoming tumor immune evasion mechanisms [339]. Additional optimization efforts yielded PVTX-405, a tetracyclic spiro-iso-indolinone-based IKZF2 molecular glue degrader with enhanced oral bioavailability and superior pharmacokinetic profiles across multiple animal models [340]. Zhang and colleagues subsequently identified compound 55, an iso-indolinone-based glutarimide, which achieves profound and sustained IKZF2 degradation in murine lymphoid tissues following oral administration, outperforming established benchmarks in both selectivity and solubility [341].

Methodologically, Dudas et al. established a pathway-agnostic thermodynamic framework for quantifying cooperativity (ΔG_coop) and implemented an integrated computational pipeline combining molecular docking, dynamics simulations, and free energy perturbation calculations [342]. This approach enables virtual screening of large chemical libraries to identify novel degrader candidates with potency exceeding known references, thereby creating new opportunities for targeting transcription factors traditionally considered undruggable.

A persistent challenge in the field remains the discrimination between direct degradation events and indirect downstream effects. To address this, a proteome-wide mass spectrometry methodology (DegMS) was developed. This method specifically excludes confounding signals from transcriptional and translational adaptations following target depletion [343]. Validation studies demonstrated DegMS’s capability to identify direct targets of cyclin K degraders dCeMM2/4 and the GSPT1-directed compound CC-885. Notably, the application of this methodology revealed the zinc finger protein FIZ1as a previously unrecognized degradation target, highlighting its potential for the systematic discovery of novel degrader targets [343].

TPD, despite its transformative potential in eliminating pathogenic ZFPs, still confronts significant challenges. These include the ongoing development of rational design principles, especially for molecular glues that currently depend heavily on empirical screening [344]. Additional hurdles involve achieving tissue-specific delivery and overcoming potential resistance mediated by on-target mutations or adaptive E3 ligase responses [345]. Future directions encompass expanding the E3 ligase toolbox, integrating artificial intelligence with computational methods, and extending applications to non-oncological domains, including neurodegenerative and autoimmune disorders. As these challenges are progressively addressed, targeted protein degradation continues to advance toward providing transformative therapeutics for ZFP-related pathologies. Concurrently, a more fundamental therapeutic paradigm is gaining momentum—the direct editing or regulation of the genome itself. This progression from proteome- to genome-level intervention marks the next frontier in the definitive treatment of ZFP-driven pathologies.

### Gene editing and gene regulation

Beyond direct pharmacological targeting of endogenous ZFPs with small molecules, strategies have emerged that integrate the tunability of small molecules with the targeting precision of engineered ZFPs, thereby enabling more refined therapeutic interventions. For example, researchers constructed a mifepristone-controlled “molecular switch” by fusing a ZFP DNA-binding domain, specific to the *VEGF-A* gene, with a drug-inducible regulatory domain. This system activates endogenous gene transcription only upon drug administration, which enables dose-dependent and reversible modulation of gene expression [346]. This approach strategically combines the pharmacological properties of small molecules with the precision of gene regulatory tools, thus advancing a more programmable therapeutic paradigm for the foundational manipulation of ZFPs and their regulatory networks through gene editing and gene regulation technologies.

Gene editing, exemplified by zinc finger nucleases (ZFNs), aims for permanent genomic modification. ZFNs utilize customized zinc finger arrays to bind specific DNA sequences and employ the *FokI* endonuclease to introduce double-strand breaks. Cellular mechanisms then repair these breaks, facilitating targeted gene knockout or correction [347]. This approach has been effectively applied in disease models to establish direct mechanistic-therapeutic linkages. For instance, in PD models, ZFN-mediated correction of the leucine-rich repeat kinase 2 (LRRK2) G2019S mutation restored kinase activity, thereby reversing downstream pathological cascades including ERK signaling dysregulation, autophagy deficits, and pathogenic phosphorylation of α-synuclein, which ultimately rescued synaptic morphology and stress resilience in dopaminergic neurons [348]. Similarly, in Angelman syndrome, a ZFN pair designed to target 86 repetitive Snord115 genes within the ubiquitin protein ligase E3A antisense transcripts (UBE3A-ATS) silencing element achieved multi-locus editing, which durably relieved epigenetic repression of the paternal UBE3A allele and restored protein expression in vivo, demonstrating a multi-target intervention strategy [349]. These applications establish a paradigm wherein molecular intervention is directly linked to functional recovery. Importantly, the capacity for coordinated multi-target editing, as demonstrated in these models, provides a direct proof of concept for the treatment of complex, multi-factorial diseases such as ischemic stroke. In this context, ZFNs could be engineered to simultaneously modulate key nodes across distinct yet interconnected pathological networks, including those mediating inflammation, oxidative stress, and apoptosis, thereby enabling synergistic therapeutic outcomes. However, the design and assembly of highly active ZFNs remain technically challenging. Recent advances, including rational design assisted by computational tools such as AlphaFold and Rosetta, have improved editing efficiency by approximately 5%, underscoring the role of structural biology in optimizing these editors [350].

Building on these technological foundations, ZFN-based editing has demonstrated therapeutic potential across a range of diseases, with applications advancing from preclinical to clinical stages. Notable in vivo approaches include single intravenous infusion of ZFN-encoding vectors to target hepatocytes, where detectable liver editing is linked to transient increases in plasma enzyme activity, as demonstrated in clinical trials for mucopolysaccharidosis [351]. In vitro strategies have been clinically applied to hematopoietic stem cells to disrupt the BCL11A enhancer for fetal hemoglobin reactivation in sickle cell disease [330], and to edit the CCR5 gene in CD4⁺ T cells for HIV resistance [352]. However, broader clinical translation faces safety challenges, primarily concerns over off-target editing effects and potential immunogenicity against the engineered nucleases or delivery vectors, which could impact therapeutic efficacy and long-term safety [353]. These challenges are being actively mitigated through continual refinements to nuclease architecture and advanced delivery systems, such as nanoparticle shielding [354, 355].

This technological advancement also provides support for the discovery and validation of novel therapeutic targets. For instance, in Parkinson's disease research, the pathogenic *LRRK2 G2019S* mutation has been precisely corrected in models through ZFN-mediated homology-directed repair [348]. Concurrently, genome-wide association studies have further revealed that genetic variations in ZFP family genes are also associated with disease risk. For example, multiple loci in *ZFP208* (such as rs2188971, rs2188972, rs8103163, and rs7248488) are significantly associated with the risk of ischemic stroke [356]. Collectively, these findings indicate that both well-defined pathogenic gene mutations and genetic variations in regulatory ZFP genes can serve as potential targets for ZFN-mediated genome editing, offering an important direction for precision medicine based on individual genetic backgrounds.

In contrast to permanent editing, gene regulation strategies focus on the reversible fine-tuning of gene expression. These approaches may harness endogenous epigenetic mechanisms to silence or activate ZFP genes, such as *ZFP334*, *ZFP671*, *ZBTB18*, and *ZEB1* [60, 204–206]. Alternatively, they may utilize endogenous ZFPs, including MTF-1 and SP1, which function as natural transcriptional effectors in cellular signaling pathways [234, 235, 252]. More advanced strategies involve the design of artificial ZFP transcription factors, including the drug-regulated systems described previously [346]. The therapeutic utility of these systems has been validated in whole-organism models. For example, a synthetically engineered ZFP successfully induced *VEGF-A* expression in vivo, promoting physiological angiogenesis and accelerating wound healing without inducing the vascular hyperpermeability often associated with conventional cDNA overexpression [357]. In models of traumatic brain injury, adenovirus or adeno-associated virus (AAV)-delivered *VEGF*-targeted ZFP transcriptional activators reduced neuronal apoptosis, improved synaptic function, facilitated vascular repair, and enhanced neurofunctional recovery during both acute and chronic phases [358].

In summary, gene editing, including ZFNs, and gene regulation represent two fundamental strategies for intervening at the root level of ZFP function. Gene editing is suited for definitive genetic correction, whereas gene regulation, spanning from endogenous mechanisms to artificial transcription factors, offers reversible and dynamic control, making it ideal for managing complex disease processes. It is noteworthy that ZFNs, as a first-generation programmable gene-editing tool, face challenges related to design complexity, screening efforts, cost, and intellectual property constraints. The subsequent advent of simpler, more cost-effective, and versatile CRISPR-Cas9 systems has shifted research priorities considerably. While CRISPR-Cas9 generally offers superior editing efficiency and targeting flexibility, thereby diminishing the broader use of ZFNs [359, 360], ZFNs retain specific practical advantages for therapeutic applications, particularly in neurological disorders. These include their relatively compact size, which facilitates efficient delivery via AAV vectors—a cornerstone of in vivo gene therapy for the central nervous system due to AAV's neuronal tropism and limited cargo capacity. Additionally, the expiration of their core patents may lower barriers to development and encourage their tailored application in niche neurological indications [350].

### Delivery system optimization

The development of efficient and safe delivery systems is critical for the successful clinical translation of ZFN-based gene therapies. Current strategies are broadly categorized into viral and non-viral approaches, each offering distinct advantages and playing complementary roles. Viral vectors remain a classical method for delivering ZFN-encoding genes. Among these, AAV has been extensively investigated due to its favorable safety profile and low immunogenicity [361]. For example, self-complementary AAV (scAAV) vectors delivering ZFNs designed to target the HBV polymerase demonstrated complete suppression of HBV DNA replication by inducing site-specific mutations, with minimal off-target effects observed, thereby highlighting their therapeutic potential [362]. In models of CNS disorders, AAV-mediated ZFN delivery successfully reduced specific pathogenic protein levels without impairing neuronal function, further validating this approach [363]. Clinical experience with AAV vectors has already been established for other genetic conditions, such as mucopolysaccharidosis type II (MPS II) [351]. However, viral delivery faces challenges related to limited packaging capacity and pre-existing or induced immune responses. To circumvent packaging constraints, researchers have utilized *T2A* peptides to link two ZFN monomers, which enables their co-expression from a single transcriptional cassette while preserving editing efficiency [364]. Techniques such as stereotactic intracerebroventricular (ICV) injection also facilitate precise, localized delivery to target tissues [365].

A significant limitation of viral vectors, particularly AAV, is their tendency to elicit neutralizing antibodies, which complicates strategies involving repeated administration to enhance editing efficiency. To address this, non-viral delivery systems, especially lipid nanoparticles (LNPs), have attracted considerable interest due to their low immunogenicity and suitability for repeat dosing. Studies indicate that LNP-encapsulated mRNA encoding ZFNs achieved more than 90% target gene knockout in mice at very low doses [359]. Moreover, combining ZFN mRNA-LNPs with a promoterless AAV donor template carrying a therapeutic gene enabled efficient targeted integration and sustained therapeutic protein expression. Repeated administration of the ZFN mRNA-LNPs further increased editing and expression levels significantly, which offers a novel strategy for diseases requiring multiple treatments [359]. Beyond nucleic acid delivery, direct protein-based delivery is also being explored, exemplified by chimeric peptide systems such as eTAT-ZF9-NLS, which facilitate efficient cellular transfection of ZFN proteins or their encoding plasmids [366].

In summary, clinical progress in ZFN-based therapies is fundamentally reliant on advances in delivery technology. Sustained tissue-specific gene expression can be achieved with viral vectors, notably AAVs, although their utility is constrained by immunogenicity and limited cargo capacity. These shortcomings have accelerated the development of non-viral platforms such as LNP-mRNA complexes, whose capacity for repeat dosing and highly efficient transient editing provides a powerful complementary strategy. Collectively, these evolving delivery systems are enhancing the efficacy and safety profile of gene-editing therapies. With the progressive resolution of delivery constraints, the next critical challenge lies in advancing the discovery and design of interventional tools that target ZFPs, encompassing both small molecules and engineered proteins. This imperative has catalyzed the development of integrated screening and engineering strategies, which now systematically incorporate computational design and deep learning.

### Screening and engineering strategies

The protective functions of ZFPs across various diseases establish them as highly promising therapeutic targets. However, their translation into clinical therapeutics encounters a fundamental challenge: ZFPs have long been considered “undruggable” due to their typical lack of well-defined, ligand-binding pockets. To overcome this barrier, engineering strategies that integrate computational design and deep learning have become a key driving force.

First, structure-based virtual screening offers a direct pathway for discovering small molecules that target ZFPs, an approach validated at multiple levels. At the domain level, a proof-of-concept study targeting the zinc finger domain of USP5 successfully identified high-affinity lead compounds, which confirmed the principle of this strategy [367]. At the full-protein level, a comprehensive pipeline targeting the transcription factor ZFP726 identified a lead molecule (zoledronic acid monohydrate) via molecular docking, which reversed disease phenotypes in cellular models [368]. This collective evidence robustly supports the application of virtual screening, providing a translatable template from in silico discovery to in vitro validation, and thereby accelerating small-molecule therapeutic development against ZFPs. Given that many ZFPs indeed lack readily targetable sites, phenotype-based screening and functional modulation represent another crucial complementary strategy. This approach seeks compounds that modulate ZFP expression or function rather than directly inhibiting activity. For example, a recent study systematically mined comparative toxicogenomic databases to identify 108 candidate compounds capable of activating KLF4, a key therapeutic target in stroke [369]. This work provides valuable chemical probes and starting points for drug development targeting ZFPs that resist direct inhibition.

Second, significant progress has been made in engineering ZFPs as programmable gene regulatory tools. These advancements include the establishment of a universal purification platform to overcome bottlenecks in large-scale preparation [370] and the development of deep learning models, such as *ZFDesign*. This model trained on massive protein–DNA interaction data, which enables the precise de novo design of zinc finger transcription factors for any genomic locus [371]. The reliability of such designs is underpinned by mechanistic insights into the dynamics of ZFP-DNA binding. Studies demonstrate that inter-finger linker regions and specific residues (e.g., lysine) are critical for precise positioning and for maintaining essential interactions with the DNA backbone [372]. To further ensure therapeutic safety, computational models like *ZFP-CanPred* can predict the functional consequences of ZFP mutations, effectively distinguishing pathogenic from neutral variants, thereby mitigating development risks [373]. This integrated computational framework, combining design with safety prediction, therefore aims to ensure that engineered ZFPs achieve precise genomic targeting while minimizing off-target effects. This approach, which integrates gene editing and expression regulation capabilities, effectively addresses key constraints of CRISPR-based systems, including their large size complicating delivery, pre-existing immunogenicity, reliance on protospacer adjacent motif (PAM) sequences, and non-native effector contexts. Specifically, *ZFDesign* circumvents these limitations by enabling compact, human-derived ZF arrays compatible with AAV delivery, allowing PAM-independent targeting, and supporting the seamless reprogramming of endogenous transcription factors to preserve native regulatory context [371]. For instance, to enhance specificity, ZFPs can be fused to epigenetic modifiers to achieve site-specific epigenetic editing without DNA cleavage. The therapeutic feasibility of this approach has been demonstrated in mouse models via lipid nanoparticle-mediated mRNA delivery [374].

In summary, therapeutic strategies targeting ZFPs are advancing synergistically along two primary avenues: small-molecule discovery and ZFP engineering. The first involves actively exploring small molecules that modulate the function of traditionally “undruggable” ZFPs through structure- and phenotype-based screening. The second entails engineering ZFPs themselves into programmable, highly specific gene regulatory and editing tools using deep learning and computational biology. These two pathways are complementary, thereby collectively expanding the potential applications of ZFPs in precision medicine. Looking forward, the continued integration of protein design, delivery technologies, and artificial intelligence models promises to accelerate the transition of more efficient and safer ZFP-targeted therapies from concept to clinic, ultimately offering novel solutions for treating associated diseases.

## Conclusions and future perspectives

ZFPs, the largest and most functionally diverse superfamily of transcriptional regulators in eukaryotes, are now recognized as central players in the control of gene expression and cellular fate. Under physiological conditions, they function as essential “molecular switches,” directing fundamental processes such as cellular development, differentiation, metabolic homeostasis, and DNA damage repair through sequence-specific interactions with DNA, RNA, or proteins. Conversely, their dysregulation, manifested through aberrant expression, gain- or loss-of-function mutations, or disruption of transcriptional networks, frequently drives a wide spectrum of pathologies, including cancer, CNS disorders, genetic disorders, autoimmune diseases, and viral infections. Consequently, deciphering the precise functions and regulatory logic of ZFPs across diverse physiological and pathological contexts is fundamental to unraveling biological complexity and developing novel therapeutic approaches.

Based on their central biological role, intervention strategies targeting this traditionally “undruggable” class of proteins are now undergoing methodological breakthroughs. Traditional approaches, including small-molecule screening and structure-based virtual design (e.g., targeting ZFP726), continue to yield lead compounds that directly modulate ZFP activity [367, 368]. More innovatively, targeted protein degradation technologies, such as PROTACs and molecular glues, enable the selective elimination of pathogenic ZFPs (e.g., IKZF2) by recruiting E3 ubiquitin ligases, thereby offering a route to neutralize previously intractable targets [338–341]. In parallel, gene editing and regulatory tools, including ZFNs and programmable artificial transcription factors, allow not only permanent genomic correction but also spatiotemporally precise, drug-inducible control of gene expression [346, 357, 358]. Collectively, these advances signify a shift in ZFP research from functional annotation toward active intervention.

Nevertheless, translating this understanding into effective therapies confronts fundamental challenges due to the inherent biological complexity of ZFPs. Over recent decades, substantial progress has been made in classifying ZFP families, characterizing their structural domains, and defining core functions of key members such as Sp1 and ZEB1. Large-scale genomic studies have further linked numerous ZFP variants to human diseases. Despite these advances, critical knowledge gaps remain. For the majority of ZFPs, their precise in vivo functions and genome-wide transcriptional networks are still poorly defined. A central unresolved issue is their profound context-dependence: the activity and functional outcome of a single ZFP can vary substantially, sometimes opposingly, across different cell types, developmental stages, or pathological contexts. Also unclear are the mechanisms that calibrate ZFP activity to drive distinct transcriptional programs and cell fate decisions within specific cellular settings.

This contradiction underscores the central dilemma in ZFP-targeted therapy: their context-dependent, dual-role nature necessitates a critical distinction between pathological and physiological functions. For example, the same ZFP (e.g., SP1 or ZEB1) can exert opposing effects in different cellular contexts or disease stages [119, 255, 256]. Therefore, the cornerstone of therapeutic development lies in achieving context-selective modulation, which is pursued primarily through three main avenues: direct functional perturbation via small-molecule inhibitors or activators targeting specific functional interfaces; complete removal of pathogenic proteins using degradation technologies; and genetic-level intervention, either through corrective gene editing or fine-tuned regulation of expression networks using inducible artificial transcription factors. These strategies share the common goal of precisely correcting ZFP-driven pathology while sparing essential physiological functions.

Given these considerations, the translational potential of ZFP modulation extends across oncology, neurology, immunology, and genetics. In ischemic stroke, for example, where standard revascularization is limited by both a narrow therapeutic window and reperfusion injury, the temporal adaptability of ZFP-targeted therapies positions them as promising pharmacological adjuvants. Such ‘preconditioning-mimetic’ concepts may be most applicable to high-risk populations or peri-procedural settings, whereas post-reperfusion modulation would require rapid, cell-targeted delivery and careful safety profiling This example underscores a broader principle: the future clinical impact of ZFP-targeted strategies will hinge on their ability to deliver context-selective interventions within the complex pathophysiology of each disease.

Critical to realizing this vision are breakthroughs in several key domains. First, leveraging artificial intelligence and deep learning models (e.g., ZFDesign, ZFP-CanPred) will be essential for accelerating target discovery, rational drug design, and off-target risk prediction, thereby enabling the creation of more precise tools with improved safety profiles [371, 373]. Second, optimizing delivery technologies, such as AAV and LNP-mRNA systems, is critical to overcome in vivo delivery barriers and achieve tissue-targeted, repeat dosing of therapeutic agents [359, 361]. Moreover, the establishment of more sophisticated, physiologically relevant model systems is imperative to elucidate the multifunctional roles of ZFPs within complex in vivo environments, thereby informing context-specific therapeutic decisions. Ultimately, by integrating computational design, delivery innovation, and deep biological insight, ZFPs may be transformed from challenging targets into programmable therapeutic tools, paving the way for next-generation precision medicines in oncology, neurology, and beyond.

## Data Availability

Not applicable.

## Abbreviations

ZFP: Zinc finger protein
CNS: Central nervous system
AD: Alzheimer’s disease
PD: Parkinson’s disease
TFs: Transcription factors
MAZ: MYC-associated zinc finger protein
ZEB1: Zinc finger E-box binding homeobox 1
CXXC5: CXXC-type zinc finger protein 5
NuRD: Nucleosome remodeling and deacetylase
TAD: Topologically associated domain
DSBs: DNA double-strand breaks
NHEJ: Non-homologous end joining
HR: Homologous recombination
XRCC4: X-ray repair cross complementing 4
APLF: Aprataxin and PNKP like factor
PARP1: Poly (ADP-ribose) polymerase 1
BRCA1: Breast cancer type 1 susceptibility protein
BRCA2: Breast cancer type 1 susceptibility protein
m⁶A: N6-methyladenosine
ZAP: Zinc-finger antiviral protein
VSMCs: Vascular smooth muscle cells
ZEB2: Zinc finger E-box binding homeobox 1
KLF: Kruppel-like factor
IKZF: Ikaros family zinc finger
PI3K: Phosphatidylinositol 3-kinase
AKT: Protein Kinase B
mTOR: Mammalian target of rapamycin
NPCs: Neural progenitor cells
NSCLC: Non-small cell lung cancer
EMT: Epithelial-mesenchymal transition
MZF1: Myeloid zinc finger 1
GPX4: Glutathione peroxidase 4
TNBC: Triple-negative breast cancer
CRC: Colorectal cancer
HCC: Hepatocellular carcinoma
MMP: Matrix metallopeptidase
HIF-1α: Hypoxia-inducible factor-1α
TLR4: Toll-like receptor 4
NCX1: Sodium-calcium exchanger 1
HIF-1: Hypoxia-inducible factor 1
SP1: Specificity protein 1
MTF-1: Metal-responsive transcription factor 1
MT: Metallothionein
PLZF: Promyelocytic leukemia zinc finger
ZEB1: Zinc finger E-box binding homeobox 1
A20/TNFAIP3: TNF associated protein 3
MCPIP1: Monocyte chemotactic protein-induced protein-1
JNK: C-Jun N-terminal kinase
JAZF1: Juxtaposed with another zinc finger gene 1
Gli1: Glioma-associated oncogene homolog 1
I/R: Ischemia/Reperfusion
ROS: Reactive oxygen species
TIGAR: TP53-induced glycolysis and apoptosis regulator
SOD1: Superoxide dismutase 1
LncRNA: Long noncoding RNA
TRAFs: Tumor necrosis factor receptor-associated factors
TGF β1: Transforming growth factor β1
TFRC: Transferrin receptor
TJs: Tight junctions
ZFN: Zinc finger nuclease
RIPK3: Receptor-interacting serine/threonine-protein kinase 3
Nrf2: Nuclear factor erythroid 2-related factor
IL-6: Interleukin-6
YY1: Yin Yang 1
BCL11A: BCL11 transcription factor A
ALS: Amyotrophic lateral sclerosis
ICF syndrome: Immunodeficiency, centromeric instability, and facial anomalies syndrome
SLE: Systemic lupus erythematosus
MS: Multiple sclerosis
CD: Crohn’s disease
TPD: Targeted Protein Degradation
AAV: Adeno-associated virus
LNPs: Lipid nanoparticles
PAM: Protospacer adjacent motif
ZF-TFs: Zinc finger transcription factors
